# Droplet‐Based Single‐Cell Measurements of 16S rRNA Enable Integrated Bacteria Identification and Pheno‐Molecular Antimicrobial Susceptibility Testing from Clinical Samples in 30 min

**DOI:** 10.1002/advs.202003419

**Published:** 2021-02-01

**Authors:** Aniruddha M. Kaushik, Kuangwen Hsieh, Kathleen E. Mach, Shawna Lewis, Christopher M. Puleo, Karen C. Carroll, Joseph C. Liao, Tza‐Huei Wang

**Affiliations:** ^1^ Department of Mechanical Engineering Johns Hopkins University Baltimore MD 21218 USA; ^2^ Department of Urology Stanford University School of Medicine Stanford CA 94305 USA; ^3^ Division of Medical Microbiology Department of Pathology Johns Hopkins University School of Medicine Baltimore MD 21287 USA; ^4^ Electronics Organization GE Global Research Center Niskayuna NY 12309 USA; ^5^ Department of Biomedical Engineering Johns Hopkins University Baltimore MD 21287 USA

**Keywords:** diagnostics, droplets, infectious disease, microfluidics, urinary tract infections

## Abstract

Empiric broad‐spectrum antimicrobial treatments of urinary tract infections (UTIs) have contributed to widespread antimicrobial resistance. Clinical adoption of evidence‐based treatments necessitates rapid diagnostic methods for pathogen identification (ID) and antimicrobial susceptibility testing (AST) with minimal sample preparation. In response, a microfluidic droplet‐based platform is developed for achieving both ID and AST from urine samples within 30 min. In this platform, fluorogenic hybridization probes are utilized to detect 16S rRNA from single bacterial cells encapsulated in picoliter droplets, enabling molecular identification of uropathogenic bacteria directly from urine in as little as 16 min. Moreover, in‐droplet single‐bacterial measurements of 16S rRNA provide a surrogate for AST, shortening the exposure time to 10 min for gentamicin and ciprofloxacin. A fully integrated device and screening workflow were developed to test urine specimens for one of seven unique diagnostic outcomes including the presence/absence of Gram‐negative bacteria, molecular ID of the bacteriaas *Escherichia coli*, an *Enterobacterales*, or other organism, and assessment of bacterial susceptibility to ciprofloxacin. In a 50‐specimen clinical comparison study, the platform demonstrates excellent performance compared to clinical standard methods (areas‐under‐curves, AUCs >0.95), within a small fraction of the turnaround time, highlighting its clinical utility.

## Graphical abstract

Single‐cell measurements of bacterial 16S rRNA are leveraged in picoliter droplets to achieve simultaneous molecular detection of bacteria and phenotypic assessment of antimicrobial susceptibility, unlocking a 30 min sample‐to‐answer diagnostic platform for urinary tract infections.

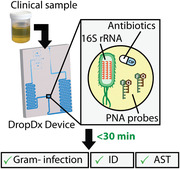

## 1 Introduction

Urinary tract infection (UTI) is one of the most prevalent infectious diseases in the world, affecting 50–60% of women at least once in their lifetime.^[^ ^1^, ^2^, ^3^ ^]^ In the United States, UTIs account for over two million emergency department visits and ≈$3.5 billion in healthcare costs annually (inflation‐adjusted to 2020).^[^ ^4^, ^5^, ^6^ ^]^ Definitive and clinically‐actionable UTI diagnostics, including both pathogen identification (ID) and antimicrobial susceptibility testing (AST), are currently based on traditional culture‐based methods and thus require several days to complete. Consequently, UTIs are often empirically treated at outpatient clinics with commonly used, broad‐spectrum antibiotics, prior to microbiological confirmation of infection or antimicrobial susceptibility. Overuse and misuse of antibiotics through such empiric antimicrobial treatment have contributed to the recent rise in antimicrobial resistant pathogens.^[^ ^7^, ^8^, ^9^ ^]^ For example, in Baltimore, USA, the resistance rate of uropathogenic *Escherichia coli* (EC) to the common broad‐spectrum antibiotic ciprofloxacin is as high as 31%, and in communities across the world, this rate is steadily increasing.^[^ ^10^, ^11^, ^12^ ^]^ The burden of increasing antimicrobial resistance is ultimately borne by the patient, as reflected by increasing UTI‐related hospitalizations, patient morbidity, and associated costs.^[^ ^6^, ^13^ ^]^

Unlike empiric treatment, evidence‐based antimicrobial treatment can significantly improve patient outcomes and curtail antimicrobial resistance.^[^ ^14^, ^15^ ^]^ However, adoption of evidence‐based treatment for UTIs in clinical settings remains contingent upon the development of a new diagnostic platform that can provide both pathogen ID and AST results well within 60 min following specimen collection.^[^ ^16^, ^17^ ^]^ Importantly, because ≈90% of uncomplicated UTIs (the most common type of UTI) are caused by the Gram‐negative species *E. coli* (≈75% of UTIs), *Proteus mirabilis* (PM), *Klebsiella pneumoniae*, and other members of the taxonomic order *Enterobacterales* (EB) (formerly members of the *Enterobacteriaceae* family^[^ ^18^ ^]^), the new platform must appropriately classify these species to establish clinical prognosis and facilitate pathogen‐specific antimicrobial therapy. Similarly, the new platform must perform AST on at least common UTI antibiotics in order to ensure appropriate therapy, safeguard these first‐line antibiotics, and support antimicrobial stewardship.^[^ ^19^, ^20^ ^]^ Finally, to operate within a clinically relevant time frame and to improve the prospect for clinical adoption, the new platform must allow direct urine sample‐to‐answer analysis without time‐consuming and labor‐intensive sample preparation steps such as pathogen isolation or nucleic acid extraction. It is therefore crucial that new UTI diagnostic platforms can simultaneously meet these important yet challenging requirements.

Recently‐developed UTI diagnostic assays and technologies have generally relied on either nucleic acid amplification tests (NAATs) for pathogen ID or phenotypic measurements for AST. Indeed, diagnostic assays based on polymerase chain reaction (PCR, the most mature of the NAATs) have become commonplace for pathogen ID^[^ ^21^, ^22^, ^23^, ^24^ ^]^ but provide little AST information. Similarly, phenotypic measurements of bacterial cells—exemplified by microfluidic‐ and single‐cell‐based strategies such as microscopy,^[^ ^25^, ^26^, ^27^ ^]^ Raman spectroscopy,^[^ ^28^, ^29^ ^]^ or microcantilevers^[^ ^30^, ^31^ ^]^—have accelerated AST to as short as <1 h but still lack molecular detection that ensures more accurate pathogen ID. These shortcomings have prompted the development of “pheno‐molecular AST”—an approach that combines quantitative, nucleic acid‐based species‐specific identification of bacteria and growth‐based (i.e., phenotypic) AST.^[^ ^32^, ^33^, ^34^, ^35^, ^36^, ^37^, ^38^ ^]^ In pheno‐molecular AST, bacteria are briefly grown in the presence and absence of antibiotics; the amounts of bacterial nucleic acids—serving as surrogates of phenotypic responses to antibiotics—are then quantitatively detected in each case and compared to reveal antibiotic susceptibilities. Pathogen ID is readily achieved via species‐specific detection probes or post‐amplification analysis (e.g., high‐resolution DNA melt curve analysis^[^ ^33^, ^34^ ^]^). To date, the most advanced pheno‐molecular AST platform can specifically detect a single pathogen (i.e., *E. coli*) and complete AST against a single antibiotic (e.g., ciprofloxacin) within 30 min.^[^ ^38^ ^]^ Though a significant advance, this platform still lacks the ability to identify or classify other prevalent uropathogenic bacteria. Furthermore, all pheno‐molecular AST platforms reported to date detect either DNA or mRNA, whose relatively low copy numbers (≈1–10 copies per gene or transcript^[^ ^39^, ^40^, ^41^ ^]^) within bacterial cells necessitate amplified detection via NAATs, and therefore must be preceded with labor‐intensive nucleic acid extraction. Thus, a rapid pheno‐molecular AST platform that can broaden the pathogen ID capacity, obviate NAATs, and minimize sample preparation still must be developed.

Ribosomal RNA (rRNA), particularly 16S rRNA, presents a promising alternative to DNA and mRNA as a molecular surrogate of phenotypic bacterial response to antibiotics in pheno‐molecular AST. Not only has 16S rRNA been well‐established for phylogenetic classification of bacteria,^[^ ^42^, ^43^, ^44^ ^]^ its abundance has been shown to correlate with bacterial growth,^[^ ^45^, ^46^, ^47^ ^]^ which suggests potential compatibility with AST. Indeed, the feasibility of 16S rRNA‐based pheno‐molecular AST has been explored, albeit relying on either cumbersome nucleic acid amplification^[^ ^37^ ^]^ or time‐consuming culture and clinical isolation.^[^ ^48^, ^49^ ^]^ We recognize, however, that the abundance of 16S rRNA (≈10^3^–10^5^ copies in a bacterial cell^[^ ^45^, ^50^ ^]^) makes it the ideal target for single‐cell detection technology based on hybridization with sequence‐specific probes in microfluidic droplets.^[^ ^51^, ^52^ ^]^ Such a detection strategy for 16S rRNA‐based pheno‐molecular AST can also readily multiplex to achieve broader pathogen ID. More importantly, this strategy can eliminate NAATs and nucleic acid extraction, thus minimizing sample preparation and enabling urine sample‐to‐answer operation.

**Figure 1:**
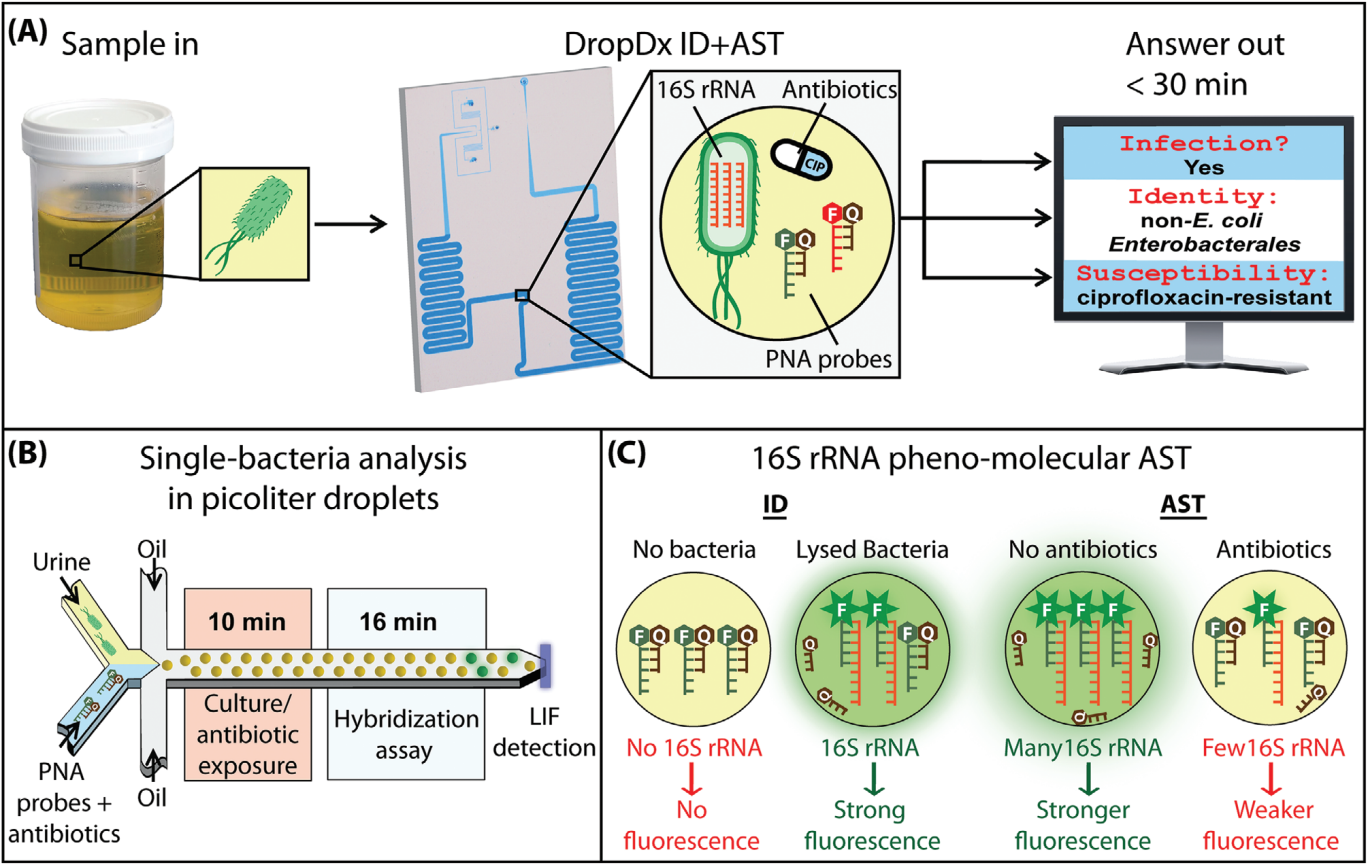
DropDx platform for rapid ID and AST of urinary tract infections. A) We have developed a urine sample‐to‐answer platform that leverages droplet microfluidics for single‐cell detection of bacterial 16S rRNA in order to confirm Gram‐negative bacterial infection, identify the causative uropathogen, and assess its antimicrobial resistance, all within 30 min of operation. B) Bacteria in urine samples are digitized into picoliter droplets along with 16S rRNA‐specific fluorogenic PNA probes and/or antibiotics before being subjected to on‐chip culture/antibiotic exposure for 10 min and probe hybridization assay for 16 min. Droplets are individually interrogated by a two‐color laser induced fluorescence (LIF) detector, and C) the fluorescence color and intensity of droplets are used to detect the presence of specific 16S rRNA sequences for identifying uropathogenic bacteria. The difference in probe fluorescence intensities between antibiotic‐dosed and antibiotic‐free droplets are used to determine the relative production of 16S rRNA in single cells, which can be used to assess the antimicrobial resistance of the pathogen.

We report herein DropDx, the first UTI diagnostic platform that uses microfluidic droplet‐based single‐cell measurements of bacterial 16S rRNA to achieve both pathogen ID and AST from urine samples in as little as 30 min (**Figure** **1**A). In DropDx, each bacterial cell in a urine sample is co‐encapsulated, within a picoliter droplet, along with an antibiotic and multiple fluorogenic hybridization probes that target 16S rRNA sequences from different uropathogenic species or phylogenetic groups. Inside these droplets, bacteria are briefly exposed to the antibiotic, then thermally lysed, and further incubated to allow hybridization between complementary hybridization probes and 16S rRNA. Upon detection, the fluorescence color of the droplet reveals the bacteria's ID and the fluorescence intensity of the droplet, which is proportional to the quantity of 16S rRNA within the droplet, reveals its susceptibility to the antibiotic. Such single‐cell measurements of 16S rRNA enable DropDx to achieve unprecedented diagnostic speed, requiring as little as 16 min for hybridization and fluorescence‐based ID and only 10–60 min antibiotic exposure for pheno‐molecular AST against three common antibiotics with distinct mechanisms (i.e., gentamicin, ciprofloxacin, and ampicillin). Critically, the hybridization probes endow DropDx the capacity of classifying up to nine unique uropathogenic species into appropriate phylogenetic categories that together account for ≈90% of UTI cases. As a demonstration of its potential clinical utility, we tested 50 patient urine specimens using DropDx and, in a fraction of the analysis time compared to clinical standard methods, acquired nearly identical ID and AST results (areas under curves, AUCs >0.95) and 95.3% categorical agreement.

## 2 Results

### 2.1 DropDx ID and Pheno‐Molecular AST Enabled by Single‐Cell Detection of 16S rRNA in Picoliter Droplets

In realizing DropDx, we have combined microfluidic droplet‐based single‐cell detection and 16S rRNA‐based pheno‐molecular AST into a one‐step assay within an integrated platform. To do so, we employ microfluidic flow‐focusing to confine single bacterial cells in urine into picoliter droplets along with fluorogenic peptide nucleic acid (PNA) probes. Such confinement can reduce fluorescence background from the urine sample matrix and enhance binding of probes and targets due to the increased concentration of sub‐cellular 16S rRNA from single cells, therefore reducing the turnaround time for bacterial detection. The generated droplets traverse heated regions designed to facilitate bacterial culture/antibiotic exposure for as short as 10 min and thermal lysis and PNA‐to‐16S rRNA hybridization (together, the “hybridization assay”) for as short as 16 min before being individually measured for fluorescence on‐chip by a custom two‐color laser‐induced fluorescence (LIF) detector (Figure 1B). Whereas fluorophore‐labeled PNA probes in empty droplets or droplets without target bacteria remain bound to a short quencher‐tagged complementary DNA sequence, in the presence of target bacteria, PNA probes competitively hybridize to complementary 16S rRNA targets, releasing them from their quenchers and producing a strong fluorescence signal. The fluorescence emitted from each droplet is measured using two fluorescence channels, and the resulting color signature is used to identify and classify the uropathogen (Figure 1C, “ID”). As antibiotic‐free cells produce a greater quantity of 16S rRNA after a short culture than susceptible antibiotic‐dosed cells (and comparable quantity of 16S rRNA as resistant antibiotic‐dosed cells), the difference in droplet fluorescence intensities between these two conditions can be used to assess the bacterial responses to antibiotic treatment and determine the susceptibility (Figure 1C, “AST”).

### 2.2 PNA Probe‐Based Hybridization Assay for Detection of Bacterial 16S rRNA in Urine

For designing the fluorogenic probes in our hybridization assay, we first targeted 16S rRNA from predominant UTI‐causing pathogens, and then considered its chemical structure and molecular configuration, and finally its compatibility with our LIF detector. First, we designed our probes to target *E. coli*, *P. mirabilis*, and the *Enterobacterales* order, which encompasses the two aforementioned species and other important uropathogenic genera such as *Klebsiella*, *Citrobacter*, and *Serratia*. Our EC probe, PM probe, and EB probe together account for ≈75%, ≈3%, and ≈87% of UTI cases,^[^ ^1^, ^57^ ^]^ respectively. Additionally, we designed a pan‐bacteria, universal (UNI) probe for confirming or ruling out bacterial infection, which accounts for nearly 100% of UTI cases (Table S1, Supporting Information). Next, we employed PNA in our probe because the neutrally charged polyamine backbone of PNA^[^ ^53^, ^54^, ^55^, ^56^ ^]^ is thermally stable and resistant to various pH and salt concentrations, and thus well suited for diagnostic assays in urine samples. We used a hybrid probe configuration composed of a fluorophore‐labeled PNA strand (15–17 nt) and a short complementary quencher‐tagged DNA strand (10–12 nt). Hybridization of the PNA strand to bacterial 16S rRNA target simultaneously displaces the DNA quencher strand, which liberates probe fluorescence and facilitates robust bacteria detection.^[^ ^52^ ^]^ Finally, because our LIF detector is equipped to detect FAM and Alexa546, we tagged our PNA strand with either FAM or Alexa546 fluorophores and the DNA strand with corresponding quenchers.

**Figure 2:**
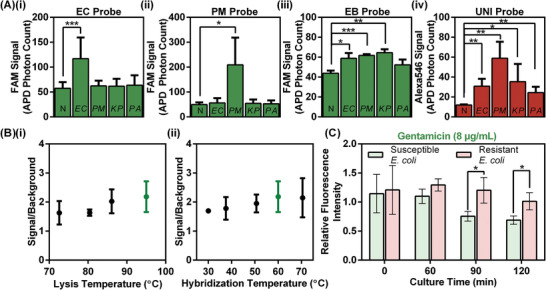
PNA probe hybridization assay for specific detection of uropathogens. A) We have designed PNA probes specific to the i) *E. coli* and ii) *P. mirabilis* species, the iii) *Enterobacterales* order, and the iv) eubacterial kingdom, in order to be able to detect all predominant uropathogens. We ensure that the designed probes can detect signal from target bacteria over blank urine background (N) by measuring probe fluorescence in the presence of *E. coli* (EC), *P. mirabilis* (PM), *K. pneumoniae* (KP), and *P. aeruginosa* (PA) (*p*‐values are calculated using unpaired one‐tailed *t*‐tests; *p < 0.05, ^**^ *p* < 0.01, ^***^ *p* < 0.001, no asterisks between bars indicates no significant difference). B) Our assay works across a wide range of i) lysis temperatures and ii) hybridization temperatures (green: selected temperatures). C) Bulk‐based pheno‐molecular AST of reference *E. coli* ATCC 25922 and multi‐drug resistant *E. coli* BAA 2471 using hybridization detection of 16S rRNA is feasible, but requires >90 min of culture/antibiotic exposure to differentiate the effect of gentamicin on the susceptible and the resistant strains of *E. coli*. Data presented as mean +/− SD, *n* ≥ 3 or *n* ≥ 2 (bulk pheno‐molecular AST).

We first verified that our probes can specifically detect target bacteria spiked into urine. In these bulk‐based (20 µL volume) experiments, we ensured that the assay conditions, including the bacterial concentration, the detection channel, and the detector, were comparable to droplet‐based detection. We spiked ≈10^9^ CFU mL^−1^ (equivalent to 1 cell in a ≈1 pL droplet) of standard reference strains of *E. coli* ATCC 25922, *P. mirabilis* ATCC 12453, *K. pneumoniae* ATCC BAA 1705, and *P. aeruginosa* ATCC 27853 into a mixture of culture‐negative (i.e., blank) urine samples and hybridization buffer with each PNA probe. Each sample was subjected to 2 min of thermal lysis at 95 °C and 30 min of hybridization at 60 °C, and then detected at room temperature within a 10 µm wide microfluidic detection channel by our two‐color LIF detector (Figure S1, Supporting Information). As expected, the FAM‐labeled EC probe (**Figure** **2**Ai) and the FAM‐labeled PM probe (Figure 2Aii) yield significantly higher signal in the presence of *E. coli* and *P. mirabilis*, respectively. The FAM‐labeled EB probe yields significantly higher signals for *E. coli*, *P. mirabilis*, and *K. pneumoniae*—all within the *Enterobacterales* order (Figure 2Aiii), while the Alexa546‐labeled UNI bacterial probe successfully detects all of the tested uropathogens (Figure 2Aiv). Of note, our PNA probe‐based assay achieves species‐specific detection in one step, without washing, and our results indicate that our PNA probes are minimally affected by nonspecific species. Additionally, using the EC probe and the reference *E. coli* strain, we also found that our assay works across a wide range of lysis temperatures (Figure 2Bi) and hybridization temperatures (Figure 2Bii). These results demonstrate that our probes are target‐specific, functional in urine, consistent at various temperatures, and can be readily implemented within droplets.

We note that we can achieve preliminary pheno‐molecular AST by directly detecting PNA‐to‐16S rRNA hybridization in the bulk format via our LIF detector, though only after relatively lengthy antibiotic exposure. To demonstrate, we incubated EC PNA probes with either multi‐drug resistant *E. coli* ATCC BAA 2471 or the reference *E. coli* strain, each strain without and with gentamicin (at a bactericidal concentration of 8 µg mL^−1^) in 20 µL sample volume for 0, 60, 90, or 120 min at 37°C before subjecting these samples to 2 min 95 °C lysis, 30 min 60 °C hybridization, and LIF detection within 10 µm wide detection channels. We then determined the relative fluorescence intensity—the ratio between the fluorescence intensities of the gentamicin‐dosed cells and the gentamicin‐free cells—for both strains for each culture duration. For the multi‐drug resistant *E. coli*, the relative fluorescence intensity remained close to 1, indicating that gentamicin‐dosed cells and gentamicin‐free cells produced similar amounts of 16S rRNA, which corroborates its resistance to gentamicin (Figure 2C, red). In contrast, for the reference *E. coli*, which is susceptible to gentamicin, gentamicin‐dosed cells were inhibited from growth and consequently produced a markedly lower quantity of 16S rRNA than gentamicin‐free cells over time (Figure S2, Supporting Information), allowing us to detect significantly lower relative fluorescence intensities compared to the multi‐drug resistant *E. coli* after 90 min (Figure 2C, green). These results confirm the feasibility of pheno‐molecular AST using our fluorogenic probe hybridization assay and pave the way for implementing the assay in droplets.

### 2.3 In‐Droplet 16S rRNA‐Based Detection of Single Bacterial Cells from Urine Samples

For achieving and characterizing 16S rRNA‐based single‐bacteria detection within droplets generated from urine samples, we established a simple urine pretreatment protocol, and we set up a modular microfluidic device (Figure S3, Supporting Information) to flexibly characterize assay time requirements. Impurities in urine samples, mostly µm to mm salt crystals, casts, and cellular debris, can clog devices and hinder droplet generation. These impurities can also autofluoresce and produce spurious fluorescence signals in droplets, obfuscating droplet analysis. Moreover, the quantity of these impurities in urine samples can vary considerably by patients and sample collection processes.^[^ ^58^ ^]^ We therefore implemented a simple protocol in which all urines samples were filtered through a gradient‐based syringe filter (iPOCdx, 35 to 2.5 µm) and diluted fourfold in MH broth prior to droplet generation and analysis (Figure S4, Supporting Information). These filtered and diluted urine samples were then co‐injected with PNA probes into the modular devices. Droplets containing single bacterial cells and PNA probes first formed in the droplet generation unit of the device, then flowed out of the device into a Tygon tubing that was placed on a 95 °C Peltier heater for bacterial lysis and a 60 °C Peltier heater for PNA‐to‐16S rRNA hybridization, and finally re‐entered the device and through the 10 µm wide detection channel, where their fluorescence signals were measured sequentially by the LIF detector (Figure S3, Supporting Information). This modular device is particularly useful for optimizing the lysis and hybridization times, as we would simply adjust the length of Tygon tubing instead of designing and fabricating new devices.

**Figure 3:**
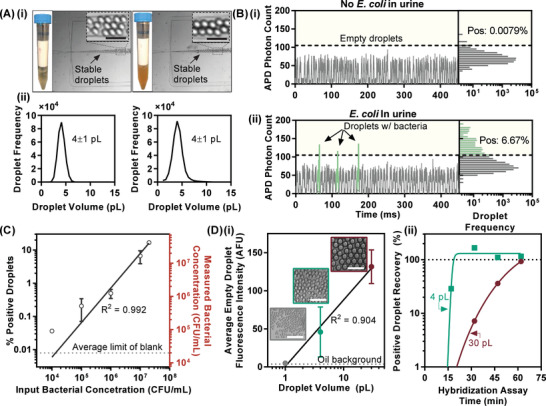
Single‐cell detection of bacterial 16S rRNA from urine using microfluidic droplets. A) i) Urine samples of distinctly different color and turbidity can be discretized using flow‐focusing to generate monodisperse droplets (scale bars ≈50 µm) of ii) 4 ± 1 pL volume. B) Droplet fluorescence peak traces i) without *E. coli*, droplets emit baseline fluorescence signal, and have a positive droplet rate of 0.0079% (also known as the average limit of blank). ii) In the presence of 10^7^ CFU mL^−1^ *E. coli*, droplets emit a higher fluorescence signal, and have a positive droplet rate of 6.67%. C) Droplet‐based quantification of *E. coli* in urine across four orders of magnitude within the clinically relevant dynamic range for UTIs (10^4^ to 2 × 10^7^ CFU mL^−1^), *R* ^2^ = 0.992 D) i) Reduction in droplet volume from 30 to 4 to 1 pL results in lower background fluorescence signals (scale bars in white ≈100 µm). ii) Compared to larger 30 pL droplets, 4 pL droplets facilitate faster generation of differentiable fluorescence signal over the reduced local background, as quickly as within 15 min. Data in (C,D(i)) presented as mean +/− SD, *n* ≥ 2 except for 2 × 10^7^ CFU mL^−1^ input bacterial concentration in (C).

We next demonstrated successful in‐droplet 16S rRNA‐based detection of single bacterial cells from urine samples. As an important prerequisite, we first showed that we could stably generate monodisperse, 4 ± 1 pL droplets from various urine samples (ranging from visibly light yellow/clear to turbid orange) without clogging (**Figure** **3**A). For demonstrating single‐cell detection, we co‐encapsulated EC probes and blank urine samples spiked without and with *E. coli* cells (10^7^ CFU mL^−1^) into droplets, heated these droplets at 95 °C for 2 min and 60 °C for 30 min, and detected these droplets using our LIF detector. The urine sample without *E. coli* yielded low‐intensity fluorescence peaks in the fluorescence peak trace that correspond to empty droplets (Figure 3Bi). In contrast, the urine sample with *E. coli* yielded additional high‐intensity fluorescence peaks in the fluorescence peak trace that indicate the presence of *E. coli* in these droplets (heretofore, “positive” droplets) (Figure 3Bii, green). Importantly, droplet fluorescence intensity histograms revealed only ≈0.0079% of (false) positive droplets (likely from autofluorescent impurities) in the urine sample without *E. coli* (Figure 3Bi) but ≈6.67% of positive droplets in the urine sample with *E. coli* (Figure 3Bii), which corresponds to ≈1.67 × 10^7^ CFU mL^−1^. The reasonable agreement between the spike‐in concentration and the measured concentration suggests that *E. coli* was indeed individually detected.

We subsequently ensured that we can detect clinically relevant bacterial loads for UTI ranging from 10^4^ CFU mL^−1^ to 2 × 10^7^ CFU mL^−1^ using our in‐droplet 16S rRNA‐based detection. Toward achieving this, our urine pre‐treatment protocol helped reduce autofluorescence in urine samples and significantly lower the limit of blank (i.e., the number of false‐positive droplets from blank urine samples), while allowing ≈94% recovery of bacteria from the samples (Figure S4, Supporting Information). Aided by the low limit of blank and high recovery of bacteria, we successfully detected 2 × 10^7^, 10^7^, 10^6^, 10^5^, and 10^4^ CFU mL^−1^ of the reference *E. coli* strain spiked in blank urine samples with high linearity (Figure 3C, *R* ^2^ = 0.992). Of note, our single‐cell droplet‐based assay was ≈5 orders of magnitude more sensitive than its bulk‐based counterpart (Figure S5, Supporting Information), which not only highlights an important advantage of in‐droplet assays, but also suggests that a similar improvement in pheno‐molecular AST can be expected.

For in‐droplet assays, reduction in droplet volume would simultaneously increase the target concentration and decrease the background noise in each droplet, thus enhancing the signal‐to‐background ratio and potentially shortening the assay time.^[^ ^59^, ^60^, ^61^ ^]^ As we aim to develop a rapid diagnostic tool, we sought to shorten the assay time by testing our assay within 30, 4, and 1 pL droplets (generated via distinct modular devices with increasingly shallow microfluidic channels). We first verified robust generation of droplets at these three volumes from blank urine samples (Figure 3Di; *n* = 3 for 30, 4 pL, *n* = 2 for 1 pL). As these no‐bacteria, empty droplets shrank from 30 to 1 pL, the average background fluorescence intensity decreased and eventually became undetectable from that of the oil phase. Because our detection method relies on calculating the percentage of positive droplets from all droplets, which necessitates accurate detection of empty droplets, we excluded 1 pL droplets from further characterization. Next, we co‐spiked *E. coli* and the EC PNA probe into blank urine samples, generated 30 and 4 pL droplets, and gradually shortened the PNA‐to‐16S rRNA hybridization time from 60 to 15 min. For each set of droplets, we compared the expected rate of positive droplets, based on the spike‐in concentration of bacteria, with the measured rate of positive droplets (“positive droplet recovery”) and found that our assay was indeed faster within 4 pL droplets, detecting the expected amount of single bacterium‐containing droplets (Figure 3Dii, 100% “positive droplet recovery”) after 30 min hybridization, compared to 60 min within 30 pL droplets. Critically, in 4 pL droplets, we could still detect single *E. coli* cells after only 15 min of hybridization (Figure 3Dii). Our focus on achieving a rapid assay, coupled with our approach of comparing the relative change between samples with and without antibiotics, prompted us to employ 15 min as the hybridization time in our in‐droplet assays hereafter.

### 2.4 Rapid Assessment of Bacterial Susceptibility to Antibiotics via Quantitative Detection of 16S rRNA from Single Cells

**Figure 4:**
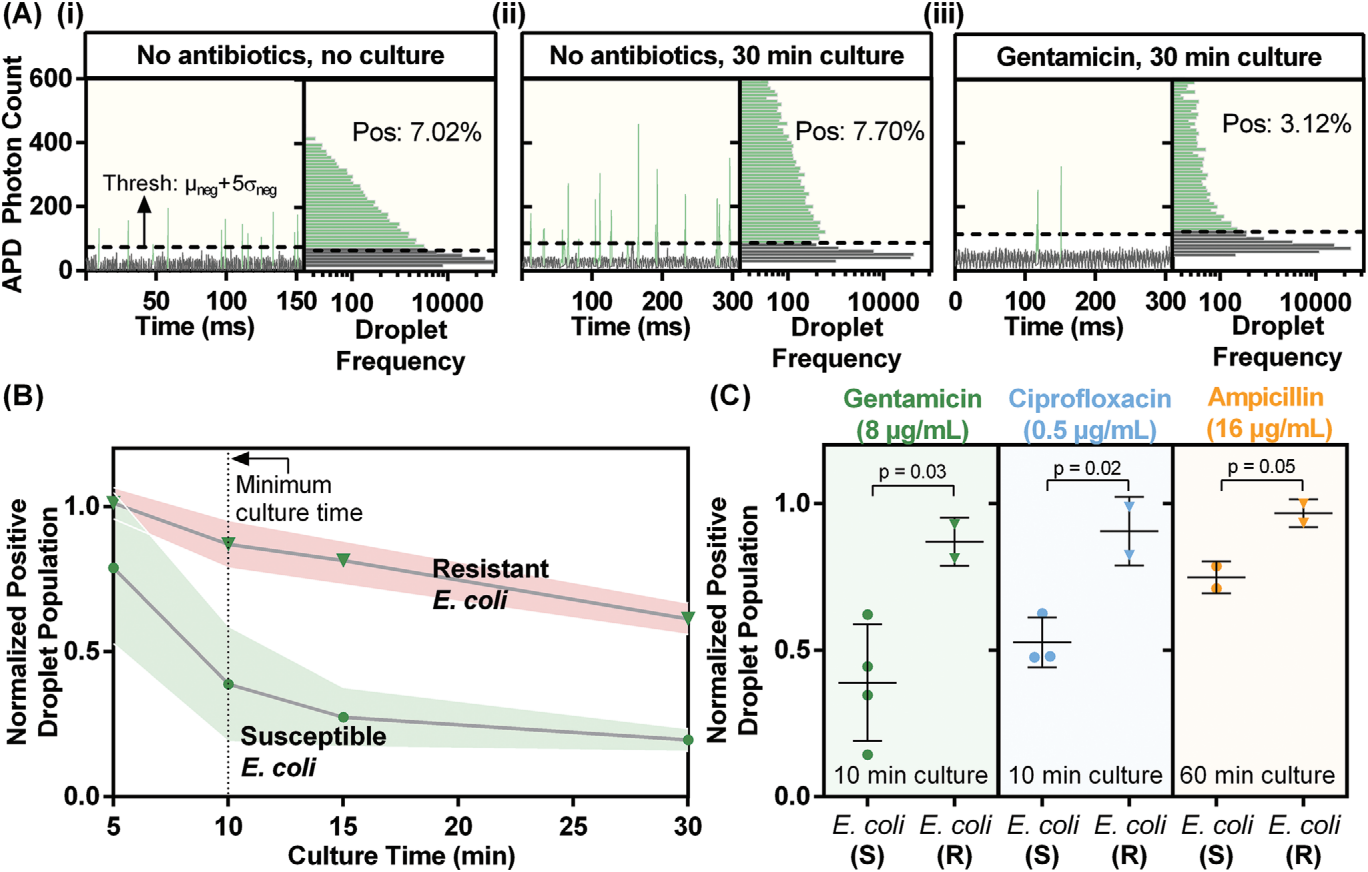
Accelerating antimicrobial susceptibility assessment via quantitative measurement of 16S rRNA from single cells. A) LIF detection of droplets containing *E. coli* cells suspended in MH broth i) without 30 min culture results in the expected 8% positive droplet frequency (7.02% observed), ii) following 30 min culture results in higher positive droplet intensities (indicative of higher 16S rRNA production) and 7.70% frequency, and iii) and after 30 min culture along with bactericidal gentamicin results in lower positive droplet intensities (indicative of relatively lower 16S rRNA production) and 3.12% frequency. B) Resistant *E. coli* can be differentiated from reference *E. coli* spiked into urine by comparing the positive droplet percentage from cells subject to antibiotic and no‐antibiotic conditions (“Normalized Positive Droplet Population”) for culture/drug exposure durations as low as 10 min. C) Resistant and susceptible strains of *E. coli* can be differentiated using our platform for three different antibiotics spanning distinct classes—gentamicin (aminoglycoside), ciprofloxacin (fluoroquinolone), and ampicillin (beta lactam). Error bars represent 1 standard deviation. The *p*‐values are calculated from unpaired one‐tailed *t*‐tests.

As initial steps toward achieving in‐droplet pheno‐molecular AST via measurements of 16S rRNA of single bacterial cells, we first demonstrated the detection of bacterial growth via increased 16S rRNA production within droplets and subsequently demonstrated inhibited bacterial growth and reduced production of 16S rRNA within droplets due to antibiotic exposure. We first co‐encapsulated the EC probe and *E. coli* cells, which were spiked into Mueller–Hinton broth (MH), into picoliter droplets. One set of generated droplets was subject to only the hybridization assay (i.e., 95 °C for 2 min for bacterial lysis, and 60 °C for 15 min for hybridization), while the other set of droplets was heated at 37 °C for 30 min to facilitate bacterial growth, before being subject to the hybridization assay. Each set of droplets were then detected with the LIF detector, and the resulting data was subject to histogram analysis, where a threshold (“Thresh,” **Figure** **4**Ai), five standard deviations above the mean intensity of empty droplets, was calculated for distinguishing positive droplets. In these experiments, the culture‐free (Figure 4Ai) and cultured *E. coli* cells (Figure 4Aii) yield frequencies of positive droplets (7.02% and 7.70%, respectively) which correspond well with the expected 8% based on the input concentration of *E. coli* (2 × 10^7^ CFU mL^−1^). Importantly, the mean intensity of the cultured *E*. *coli*‐containing droplets is much higher than that of droplets containing uncultured *E. coli*, indicative of the increased production of 16S rRNA from these growing *E. coli* cells.

Next, we co‐encapsulated the EC probe, *E. coli*‐spiked MH broth, and gentamicin (high bactericidal concentration of 128 µg mL^−1^) into picoliter droplets and performed 30 min culture/antibiotic exposure followed by the hybridization assay and LIF detection. When compared to the cultured no‐antibiotic control, the gentamicin‐dosed *E. coli* droplet population yields a relatively reduced frequency of positive droplets (3.12%, Figure 4Aiii). The reduction in positive droplet frequency can be attributed to the relatively lower average fluorescence signal from gentamicin‐dosed positive droplets. This decrease in fluorescence aligns well with our previous observations from bulk measurements (Figure S2, Supporting Information), indicating that *E. coli* cells in droplets produce lower quantities of 16S rRNA in the presence of the antibiotic (Figure 4Aiii). Indeed, the normalized positive droplet population, defined as the frequency of positive droplets with antibiotics divided by the frequency of positive droplets without antibiotics, decreases as we increase the concentration of antibiotics, indicative of an antimicrobial dose‐dependent response of bacterial 16S rRNA production (Figure S6, Supporting Information).

We next sought to minimize the culture/antibiotic exposure time for assessing antimicrobial susceptibility in urine. We separately digitized reference and multi‐drug resistant *E. coli*‐spiked urine samples, each along with 2× MH broth and EC PNA probes in droplets without and with gentamicin (bactericidal concentration of 8 µg mL^−1^). Each gentamicin‐free and gentamicin‐dosed droplet group pair was then incubated at 37 °C for 30, 15, 10, or 5 min, followed by lysis, hybridization, and LIF detection (Figure 4B). Longer bacterial culture result in higher production of 16S rRNA in gentamicin‐free bacterial cells and therefore increased positive droplet frequencies. At the same time, gentamicin restricts the production of 16S rRNA in gentamicin‐dosed reference *E. coli* cells, reflected by the decreasing normalized positive droplet population over increasing culture times (Figure 4B, green). In contrast, gentamicin has a significantly lesser effect on 16S rRNA production for the resistant *E. coli* cells, reflected by the comparatively higher normalized positive droplet population (Figure 4B, red). We note that because we measure the susceptibility with single‐cell resolution, the slight reduction in the normalized positive droplet population of resistant *E. coli* likely arises from a fraction of cells that are inhibited by gentamicin but undetectable by bulk‐based AST methods. Most importantly, the effect of gentamicin on the two strains can be differentiated after as short as 10 min culture/antibiotic exposure, the minimum time at which there is no overlap between the error envelopes around the normalized positive droplet populations. This result highlights the benefit of single‐cell measurements of 16S rRNA via droplet microfluidics, where even with drastically shortened culture times, by processing a large number of droplets at a high throughput (≈kHz), we sample sufficient single cells that are representative of the entire population to allow us to differentiate susceptible and resistant bacteria. As a result, we achieve assessment of antimicrobial susceptibility faster than the average replication time of an *E. coli* cell, a feat that ultimately paves the way for integrating molecular ID and AST to well under 30 min.

Finally, we investigated the utility of our single‐cell 16S rRNA‐based pheno‐molecular AST approach against three antibiotics from distinct classes with distinct mechanisms of action—gentamicin (an aminoglycoside that inhibits protein synthesis), ciprofloxacin (a fluoroquinolone that inhibits DNA gyrase activity), and ampicillin (a beta‐lactam that inhibits cell wall synthesis). We tested the reference and resistant strains of *E. coli* against these three antibiotics at relevant concentrations guided by the Clinical Laboratory Standards Institute (CLSI):^[^ ^62^ ^]^ 8 µg mL^−1^ for gentamicin, 0.5 µg mL^−1^ for ciprofloxacin, and 16 µg mL^−1^ for ampicillin. For both gentamicin and ciprofloxacin, our platform can differentiate antibiotic‐susceptible from antibiotic‐resistant *E. coli* after only 10 min of culture/antibiotic exposure (*p* = 0.03 for gentamicin and *p* = 0.02 for ciprofloxacin, Figure 4C). For ampicillin, although our platform also reliably differentiates the two *E. coli* strains (*p* = 0.05, Figure 4C), 60 min of culture/antibiotic exposure is required, which may be explained by the time ampicillin needs to elongate, bulge, and ultimately lyse bacteria.^[^ ^63^ ^]^ These results show the utility of our method for achieving reliable assessment of antimicrobial susceptibility for three different antibiotics with distinct mechanisms of action, though the total assay time is dependent on the antibiotic and its mechanism of action.

### 2.5 Clinical Comparison Study of DropDx for Rapid Bacterial ID Classification and Pheno‐Molecular Antimicrobial Susceptibility Assessment from 50 Clinical Urine Specimens

**Figure 5:**
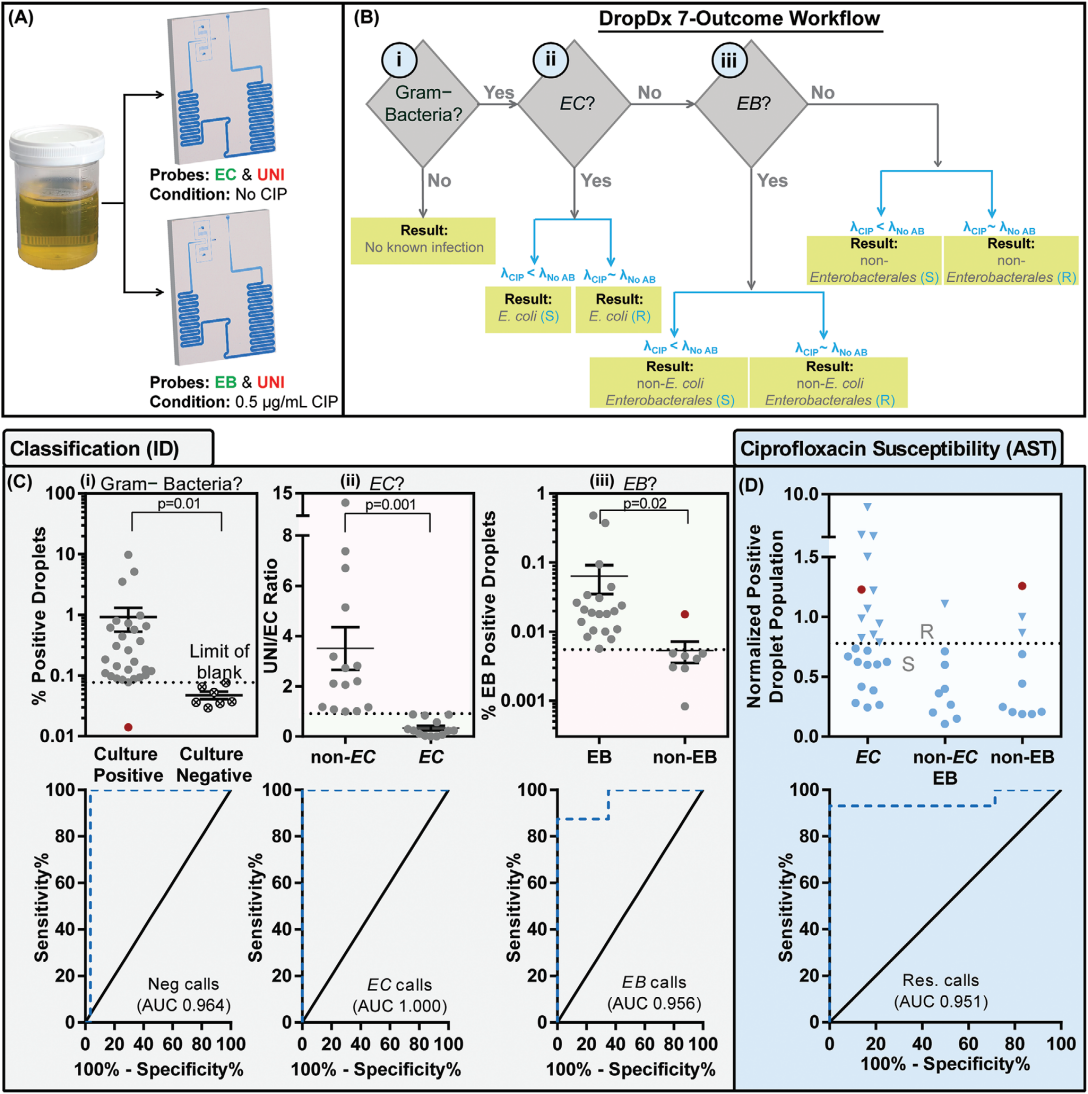
DropDx clinical comparison study of 50 deidentified patient samples from Johns Hopkins Hospital. A) Each sample was simultaneously tested using clinical standard ID/AST tests as well as with 2 DropDx devices for measurements without and with ciprofloxacin. For ID, we used a combination of EC, EB, and UNI probes. B) Our 7‐outcome DropDx workflow is used to determine if there is a Gram‐negative bacterial infection present, whether the infecting pathogen is *E. coli*, whether the infecting pathogen is in the *Enterobacterales* order, or whether the infecting pathogen is a different (Gram‐negative) bacteria and to assess the susceptibility of the infecting pathogen to ciprofloxacin. *λ* is the proportion of droplets that contain a single cell to all droplets. C) Unbiased thresholding for each diagnostic metric was conducted in pilot studies using ROC curve analysis, and the final data groups and resulting ROC curves are plotted for i) differentiating culture‐positive from culture‐negative samples (AUC: 0.964), for ii) differentiating *E. coli* from non‐*E. coli* samples (AUC: 1.000), for iii) differentiating *Enterobacterales* from non‐*Enterobacterales* samples (AUC: 0.956), and for D) differentiating ciprofloxacin resistant from susceptible samples (AUC: 0.951). Importantly, DropDx's single‐cell pheno‐molecular AST results in a categorical agreement of 95.3% with no major errors. Error bars represent mean and standard error. The *p*‐values are calculated from unpaired one‐tailed t‐tests.

To evaluate the utility of DropDx for diagnosis of UTIs, we designed a clinical comparison study by using urine specimens from patients with suspected uncomplicated UTIs—the most common type of UTI. As a prerequisite for this study, we first constructed the fully integrated platform, DropDx, for automated and hands‐free operation from sample input to bacterial diagnosis (Figure S7, Supporting Information). We designed and fabricated a monolithic DropDx device, positioned its incubation channel above three individually controlled Peltier heaters kept at either 37 °C for culture/antibiotic exposure, 95 °C for lysis, or 60 °C for hybridization (Figure S8, Supporting Information), and finally aligned its detection channel to the two‐color LIF detector for fluorescence measurements. We experimentally determined that the droplets generated in the DropDx device flowed continuously through the 37 °C segment in ≈10 min, the 95 °C segment in ≈2 min, and the 60 °C segment in ≈14 min (Figure S9, Supporting Information), resulting in an assay time of ≈26 min. We then designed a simple yet useful workflow using 2 DropDx devices, which can be performed in parallel (**Figure** **5**A), to classify seven unique UTI diagnostic outcomes that cover ≈90% UTI cases and assess susceptibility against a common broad‐spectrum antibiotic ciprofloxacin. This was accomplished by analyzing each urine specimen with FAM‐labeled EC probe, Alexa546‐labeled UNI probe, and no ciprofloxacin in the first device, and analyzing the same specimen with FAM‐labeled EB probe, Alexa546‐labeled UNI probe, and 0.5 µg mL^−1^ ciprofloxacin (the minimum inhibitory concentration interpretive breakpoint range per the CLSI guideline^[^ ^62^ ^]^) in the second device (Figure 5A). In doing so, we can determine one of seven diagnostic outcomes for each urine specimen (namely, no known infection, *E. coli* (S), *E. coli* (R), non‐*E. coli Enterobacterales* (S), non‐*E. coli Enterobacterales* (R), other (S), and other (R))—within as little as 30 min (Figure 5B). We note that we chose to test ciprofloxacin because this commonly administered broad‐spectrum antibiotic for UTIs is becoming increasingly ineffective due to emerging resistance and therefore can be safeguarded with a diagnostic tool like DropDx.^[^ ^64^, ^65^, ^66^ ^]^

Using our DropDx platform and workflow, we conducted our clinical comparison study of 50 deidentified patient urine specimens obtained from the Johns Hopkins Hospital Clinical Microbiology Laboratory (IRB00189525). Urine specimens that represent uncomplicated UTIs were selected for our study (see the Experimental Section). Each urine specimen was subject to the clinical standard diagnostic workflow that included clinical isolation via plating, ID via matrix‐assisted laser desorption/ionization–time of flight (MALDI–TOF) mass spectrometry, and AST via BD Phoenix enabled broth microdilution testing. Clinical ID/AST reports for each specimen required at least 48 h from patient collection. DropDx was used to test refrigerated urine specimens that had freshly entered the clinical workflow, in order to obtain comparable results within a small fraction of the time.

For all 50 specimens tested, DropDx delivers pathogen identification and antimicrobial susceptibility assessment results that closely agree with standard clinical results (Figure 5C; Table S4, Supporting Information). First, DropDx differentiates culture‐negative from culture‐positive specimens with high accuracy (*p* = 0.01, Figure 5Ci). The single false‐negative call is likely due to low input concentration of bacteria or high optical background from urine. Second, DropDx detects all *E. coli* from culture‐positive specimens (*p* = 0.001, Figure 5Cii), owing to the high specificity and sensitivity of the designed PNA probes. Third, DropDx successfully classifies all but 1 of the non‐*E. coli Enterobacterales* from *Enterobacterales* specimens (*p* = 0.02, Figure 5Ciii). These results correspond to excellent positive predictive values and negative predicative values (Table S5, Supporting Information). As a more robust measure of diagnostic utility, we performed receiver‐operating curve (ROC) analysis for these three metrics of classification (see the Experimental Section), which not only allowed us to determine unbiased thresholds for each metric (see the Discussion), but also resulted in ROCs with high areas‐under‐curve (AUC for Negative/Positive = 0.964, AUC for EC/non‐EC = 1.000, and AUC for EB/non‐EC EB = 0.956). Finally, DropDx accurately determines the susceptibility for 41 out of 43 culture‐positive specimens to ciprofloxacin (Figure 5D; *p* = 0.02, see Figure S10, Supporting Information). In comparing with standard AST, DropDx's single‐cell pheno‐molecular AST achieves a categorical agreement of 95.3% with no major errors, and an AUC of 0.951—a highly favorable outcome for a novel diagnostic test.^[^ ^67^ ^]^

## 3 Discussion

We introduce DropDx, the first UTI diagnostic platform that leverages droplet microfluidics for single‐cell measurements of bacterial 16S rRNA to enable unprecedented pathogen ID/classification and AST from urine in as little as 30 min. The development of DropDx was achieved by implementing three enabling concepts synergistically within an integrated platform. First, employment of 16S rRNA as a novel molecular surrogate for phenotypic bacterial response to antibiotics in the emerging approach of pheno‐molecular AST allows us to achieve both pathogen ID/classification and AST in a one‐step assay. Second, confinement of highly abundant 16S rRNA from single bacterial cells within picoliter droplets makes their detection via hybridization‐based probes possible, thus enabling rapid and simple detection of bacteria without nucleic acid amplification and minimal sample preparation. Finally, high‐throughput and quantitative measurements of 16S rRNA from hundreds to thousands of single bacterial cells via droplets offer a new and powerful approach for shortening culture/antibiotic exposure and accelerating pheno‐molecular AST.

In this work, we tailor DropDx to facilitate a useful yet practical uropathogen ID/classification scheme that can improve treatments for UTIs, especially uncomplicated UTIs, which is the most common. For pathogen ID in UTIs, rapid binary detection of a single predominant bacterial species such as *E. coli* is useful but nevertheless limited. On the other hand, broad‐based species‐level ID would undoubtedly be ideal but would require additional devices or additional assay steps, both of which can increase operational complexity and turnaround time. We instead focus on four ID/classification categories that account for ≈90% of UTI cases: whether or not a Gram‐negative bacterial infection is present and whether it is *E. coli*, in the *Enterobacterales* order, or different Gram‐negative bacteria. Because 16S rRNA is well‐characterized for determining bacterial phylogeny for species‐level, order‐level, and kingdom‐level bacterial ID, we only need three detection probes to cover the four ID/classification categories. Moreover, two‐color detection in DropDx allows us to achieve the necessary multiplexability to cover ≈90% of UTIs, using only two devices. An example case of the usefulness of our ID/classification scheme is if DropDx identifies a non‐*Enterobacterales* that is ciprofloxacin‐resistant (as with specimen #42), a physician could immediately prescribe the most appropriate alternative antibiotic based on the antibiotic resistance rates for non‐*Enterobacterales* in the community.

Our results show that, by using only the pan‐bacteria UNI probe to quantitatively measure 16S rRNA from single bacterial cells in picoliter droplets, reliable AST was achieved for nine bacterial species against ciprofloxacin. Such single‐cell measurements of 16S rRNA also achieve reliable AST for *E. coli* against gentamicin, ciprofloxacin, and ampicillin. These results suggest that, whereas multiple species‐ and/or antibiotic‐dependent mRNA sequences have been required to achieve reliable AST in other approaches,^[^ ^68^ ^]^ 16S rRNA can potentially serve as a broad surrogate for pheno‐molecular AST with less dependence on bacterial species and antibiotics. This is perhaps because 16S rRNA is an integral structural building block of ribosomes that is replicated during growth and doubling of bacterial cells, which is independent from the specific inhibitory mechanisms of the tested antibiotics. While the demonstrated combinations of bacteria and antibiotics in DropDx show great promise, further validation of 16S rRNA in pheno‐molecular AST with more bacterial species against additional classes of antibiotics (such as sulfonamides and polymyxins) is needed.

Clinically oriented evaluation of new diagnostic technologies should ideally include both a pilot phase and a validation phase that is unbiased, akin to clinical trials.^[^ ^69^ ^]^ However, such a two‐phased study design has not been a common practice for developing microfluidic‐based diagnostic technologies. We in fact have followed such a study design as we evaluated the ID and AST performance of DropDx (Figure S11, Supporting Information). Among the 50 patient urine specimens tested in this work, we used the initial 15 for AST pilot study (Figure S11, Supporting Information, Phase 1), the next 16 concurrently for ID pilot study and AST validation study (Figure S11, Supporting Information, Phase 2), and the final 19 for both ID validation study and AST validation study (Figure S11, Supporting Information, Phase 3). Of the 35 specimens in Phases 2 and 3, 28 were positive for bacterial infections and eligible for AST validation. We used the specimens in the pilot phases to 1) perform ROC analysis and find the maximum Youden's Index^[^ ^70^, ^71^, ^72^ ^]^ for determining the optimal ID and AST thresholds that would be applied, without bias, to all subsequent specimens in the validation phases, and to 2) conduct power analysis to ensure that sufficient specimens are enrolled for validation. The threshold established from the 15 specimens for calling the susceptibility/resistance to ciprofloxacin in Phase 1 was validated as it unbiasedly called the susceptibility/resistance to ciprofloxacin for 27 of the 28 eligible specimens in Phases 2 and 3. Power analysis in Phase 1 indicates that the 28 specimens tested in in Phases 2 and 3 was sufficient, as only 20 samples were required to obtain a statistical power of 90% and confidence of 95% (Figure S12, Supporting Information). Similarly, the three thresholds for calling bacterial infection, *E. coli*, and *Enterobacterales* that were established from the 16 samples in Phase 2 were validated as they successfully classified 18 out of 19 samples in Phase 3. Power analyses in Phase 2 show that statistical power of 90% and confidence of 95% for these three cases could be achieved by testing four samples in Phase 3 (Figure S12, Supporting Information), which we again exceeded. These results show that DropDx is robust enough to withstand rigorous evaluation that includes a pilot phase and an unbiased validation phase.

While DropDx represents a significant advancement for UTI diagnostics, we still see several avenues for improvements. First, we should incorporate more antibiotics into DropDx to generate a more complete antibiogram, which is useful for both diagnostics and surveillance of antimicrobial resistance. Similarly, we can enlarge our panel of fluorogenic PNA probes to broaden our pathogen ID/classification capacity. This can be accomplished by implementing LIF detectors that can detect three or four fluorescence colors or by using a multiplexed droplet device^[^ ^73^ ^]^ that can test multiple groups of droplets. We can also expand DropDx to detecting Gram‐positive uropathogens, though this would require assay modifications such as the addition of chemical lysing agents and/or cyclic thermal shocking to access their 16S rRNA or the addition of viability dyes (e.g., resazurin)^[^ ^59^, ^74^ ^]^ that non‐specifically detect any microorganism. DropDx has shown great potential for clinical utility, underscored by its amplification‐free approach and urine sample‐to‐answer workflow. As with existing commercially successful droplet microfluidic platforms (e.g., BioRad Technologies, RainDance Technologies), engineering a simpler user interface by miniaturizing and packaging the fluidic and optical components can further improve the clinical deployability of DropDx. Ultimately, successful implementation of these improvements will further empower DropDx to facilitate evidence‐based antimicrobial treatment, improve patient care for increasingly life‐threatening infectious diseases, and allay the threat of antimicrobial resistance.

## 4 Experimental Section

##### Reference Bacteria, Antibiotics, and Urine Samples

Reference strains of *E. coli* (ATCC 25922), *P. mirabilis* (ATCC 12453), *K. pneumoniae* (ATCC BAA 1705), *P. aeruginosa* (ATCC 27853) as well as a multi‐drug resistant strain of *E. coli* (ATCC BAA 2471) were all purchased from ATCC (Manassas, VA). Of note, the multi‐drug resistant strain is reported to be resistant to a variety of drug classes including carbapenems, fluoroquinolones (e.g., ciprofloxacin), *β*‐lactams (e.g., ampicillin), and aminoglycosides (e.g., gentamicin). All bacterial strains were individually plated, and an isolated colony from each was grown until log phase in tryptic soy broth (TSB). The bacteria were then counted via plating in tryptic soy agar (TSA), and stocks were aliquoted and frozen with 20% glycerol (v/v) at −80 °C. Prior to each experimental run, a fresh aliquot of bacteria was thawed and washed twice with Mueller–Hinton II cation‐adjusted broth (MH) (Sigma‐Aldrich, St. Louis, MO, USA). During experiments, bacteria were suspended in either MH or a mixture of 2× MH and culture‐negative urine at twice the specified concentrations, before being diluted twofold on‐chip by the probe solution. For all droplet experiments, the on‐chip bacterial concentration never exceeded 2 × 10^7^ CFU mL^−1^, which ensured that the probability of encapsulating two or more cells within a droplet was negligible (at most 0.3%).^[^ ^59^, ^75^ ^]^

##### Design and Preparation of PNA Probes

PNA probes were designed to target specific regions in the 16S rRNA for organisms belonging to the EB order, the EC and PM species, and the eubacterial kingdom (UNI), based on the previous work^[^ ^52^, ^76^ ^]^ (Table S1, Supporting Information). The EB, EC, and PM probes were labeled with a FAM fluorophore, whereas the universal probe was labeled with an Alexa546 fluorophore. For each PNA probe, a short complementary DNA quencher was designed labeled with an Iowa Black dark quencher. All probes were synthesized by PNA Bio (Newbury Park, CA), and all DNA quencher strands were synthesized by Integrated DNA Technologies (San Diego, CA). Probes and quenchers were stored in a custom hybridization buffer consisting of 50 × 10^−3^ m NaCl, 25 × 10^−3^ m Tris–HCl pH 8.0, and 0.5% polyvinylpyrrolidone (PVP) at −20 °C. PVP was included to reduce the likelihood of probes being adsorbed to or retained by the walls of the PDMS devices. Prior to each experiment, individual aliquots of PNA probes were first thawed at 60 °C for 10 min to ensure full dissolution of the PNA stock into the hybridization buffer. For both bulk‐based and droplet experiments, 200 × 10^−9^ m PNA probes were mixed with 600 × 10^−9^ m of the corresponding quencher in the custom hybridization buffer, and this solution was either mixed in bulk or co‐encapsulated into droplets at a 1:1 ratio along with bacteria‐containing urine and/or MH suspensions. An appropriate dosage of antibiotics was included in this mixture if required for the experiment.

##### Design and Fabrication of Microfluidic Devices

Three distinct microfluidic devices were used in this work—two for assay characterization and one for clinical testing and validation of the DropDx workflow. The first microfluidic device was used for bulk‐based characterization of the PNA probe assay, and consisted of an array of single channels, each with a 10 µm wide × 20 µm tall constriction for LIF detection of bulk assay reactants (Figure S1, Supporting Information). For assay characterization in droplets, a droplet device was designed that consisted solely of a droplet generation module and a droplet detection module. The two modules were connected by Tygon tubing of variable length, which enabled testing of varying hybridization durations (Figure S3, Supporting Information). This device consisted of a 10 µm wide × 20 µm tall flow‐focusing nozzle for droplet generation and an equidimensional constriction for droplet detection. Casting molds for both characterization devices were constructed by spinning a 20 µm layer of SU8‐3050 onto a 4 in. silicon wafer and patterning using standard photolithography. In order to test larger and smaller droplet volumes, the height of the droplet generation module was increased or lowered appropriately by spinning a thicker or thinner layer of SU8‐3050 photoresist.

Following assay characterization, an integrated DropDx device was designed that consists of a droplet generator capable of producing 4 pL droplets, a culture/drug exposure channel that houses droplets for 10 min, a lysis channel that houses droplets for 2 min, a hybridization channel that houses droplets for 14 min, and a droplet detection constriction. The DropDx device consists of a 10 µm wide and 20 µm tall flow‐focusing nozzle for droplet generation, followed by a 1000 µm wide × 60 µm tall × 169 mm long serpentine incubation channel for bacterial culture/drug exposure, a 1000 µm wide × 60 µm tall × 33 mm long channel for bacterial lysis, and a 1000 µm wide × 60 µm tall × 248 mm long channel for probe hybridization. Finally, the channels narrow into a 10 µm wide and 20 µm tall droplet detection window before widening back into an outlet. A casting mold was fabricated by first spinning a 20 µm layer of SU8‐3050 photoresist (MicroChem) onto a 4 in. silicon wafer and patterning only the droplet generation and droplet detection regions using standard photolithography. Next, a 60 µm layer of SU8‐3050 was spun onto the same mold in order to create the three incubation regions. The photomask of the incubation channels was aligned to the mold using microscopy in order to ensure continuity from droplet generation to droplet detection. Patterning of the second layer was completed using standard photolithography.

All microfluidic devices were created by pouring 30 g of 10:1 ratio of polydimethylsiloxane (PDMS) Sylgard 184 (Dow Corning) base to curing agent onto the silicon/SU8 mold. After curing the PDMS replica, holes were punched onto the device inlet ports and the device outlet port. The PDMS replica was then permanently bonded to cover glass (130 µm thickness, Ted Pella) through oxygen plasma treatment in order to seal the channels. Prior to device operation, all microfluidic chips were treated with Aquapel and baked at 80 °C for at least 20 min to render microfluidic channel surfaces hydrophobic.

##### Operation of Microfluidic Platform

One‐step pretreated urine samples (≈20–50 µL) (Figure S4, Supporting Information) and 50 µL probe/quencher mix (without or with antibiotics) were first both manually drawn via syringes into two separate ≈25 cm long sections of Tygon tubing. Both sections of Tygon tubing (sample and probes) were carefully connected to separate Hamilton 1000 glass syringes (Sigma‐Aldrich) containing FC‐40 oil (Sigma‐Aldrich), eliminating any air gaps at the interface of the aqueous and oil phases. FC‐40 oil in the syringe was used to push the aqueous phases from the Tygon tubing into the device using a syringe pump (Harvard Apparatus). BioRad QX200 Droplet Generation Oil (BioRad Laboratories) was introduced into the oil inlet of the device by a separate syringe pump. A flow rate of 15 µL h^−1^ was used for both aqueous phases, while 60 µL h^−1^ was used for the oil phase. The flow rates were chosen to ensure stable droplet generation and uniform droplet volume throughout each experiment (Table S2, Supporting Information). In order to confirm stable and uniform droplet generation, the device was imaged using a 4× objective lens and a CCD camera during droplet generation and after droplet incubation (Video S1, Supporting Information). In the modular device, generated droplets flowed through Tygon tubing connecting the droplet generation and droplet detection regions of the microfluidic device (Figure S3, Supporting Information). Importantly, separate lengths of this incubation Tygon tube were clamped onto a 95 °C Peltier heater and a 60 °C Peltier heater, such that all droplets could flow through the lysis and hybridization regions for the appropriate duration required for the experiment. Droplets traveling at 60 µL h^−1^ traversed 1 cm through the Tygon tube every 2 min. Thus, to ensure 2 min lysis and 30 min hybridization, 1 cm of the Tygon tubing was clamped to the 95 °C heater and 15 cm of the tubing to the 60 °C heater. In a similar manner, the incubation channels of the integrated DropDx device was affixed to 3 Peltier heaters set to a 37, 95, and 60 °C, respectively. A schematic containing all the major components of the microfluidic platform, including tubing, syringe pumps, device, heaters, and LIF detector is provided in Figure S7 in the Supporting Information.

##### Droplet Fluorescence Detection

Continuous‐flow droplet measurements in two colors was conducted using the LIF detector that consists of an optical stage which is interfaced with 488 and 552 nm laser excitation sources (OBIS, Coherent, Inc., Santa Clara, CA) and two silicon avalanche photodiode detectors (APD) (SPCM‐AQRH13, Excelitas Technologies, Waltham, MA). The 488 nm laser was operated at 4 mW power and the 552 nm laser was operated at 10 mW power to ensure sufficient illumination and acceptably high signal over background from each fluorophore (Table S3, Supporting Information). Both lasers were focused into the detection zone of the device using a 40× objective (Thorlabs RMS40X‐PF, NA 0.75, focal depth ≈0.6 µm). As droplets flowed through the custom laser‐induced fluorescence detection zone, fluorescence data was continuously acquired and recorded using the APDs with 0.1 ms sampling time. Each droplet flowed through the detection region every ≈1 ms, allowing for a maximum detection rate of ≈1000 Hz. Because the droplet generation rate in the device was ≈333 Hz, individual fluorescence peaks corresponding to individual droplets as well as gaps between droplets could be clearly observed. A custom LabVIEW program was used to display and save APD measurements in real time.

A custom MATLAB program was developed for analyzing the raw fluorescence intensity data acquired from the APDs. From the fluorescence intensity time trace of each experimental run, the program looks for individual droplets by quantifying peak widths and peak heights. Once droplet position and fluorescence intensity were identified for all droplets in a sample, the intensities were plotted as a histogram with ≈150 bins. Resulting histograms typically followed a bimodal distribution, and in order to classify the respective subpopulations, the first droplet histogram peak (“empty droplets”) was fit with a Gaussian curve using a Gaussian mixture model in MATLAB. Based on the fitted curve, the mean intensity of the empty droplet population as well as the standard deviation was calculated. All droplets whose intensities were at least five standard deviations above the empty droplet mean intensity were counted as bacteria‐containing “positive droplets.” Signal to background ratios were calculated by dividing the mean intensity of positive droplets by the mean intensity of empty droplets.

##### Statistical Analyses and Curve Fitting

All reported *p*‐values in this work were calculated by performing unpaired one‐tailed *t*‐tests in GraphPad Prism (GraphPad Software Inc., San Diego, CA). GraphPad was also used to fit curves for droplet data in Figure 3C,D and for extracting relevant fit parameters.

##### Validation of DropDx Platform with Clinical Urine Specimens

Fifty UTI specimens were obtained and tested under an approved institutional review board (IRB) study at the Johns Hopkins University School of Medicine (JHU‐SOM) (IRB00189525). All tested specimens, obtained from the Johns Hopkins Hospital (JHH) Clinical Microbiology Laboratory, were also subject to the standard clinical specimen management workflow. In the standard workflow, 1 to 10 µL of each specimen was plated in order to achieve clinical isolation of the uropathogen, and the remaining urine specimen was refrigerated. Typically, within 8 h of plating, the relative uropathogenic load can be estimated by direct observation of the plate. For the evaluation of DropDx, which focused on uncomplicated UTIs, refrigerated urine specimens were selected that represented typical uncomplicated UTIs,^[^ ^1^, ^77^ ^]^ or those with an estimated load of >30 000 CFU mL^−1^ of a single dominant uropathogen. This screening process was implemented specifically for the clinical comparison study, which focused on evaluating DropDx and establishing the novel method as opposed to being a large‐scale clinical study. With such screening, the platform could be tested against different sample types instead of analyzing a large amount of culture‐negative samples or samples with a low level of flora below the established clinical threshold for UTI‐positive infection. At JHH, each clinically isolated bacteria specimen was identified using MALDI–TOF mass spectrometry (Bruker Daltonics, Inc., Billerica, MA) and subject to AST using BD Phoenix (BD Diagnostics, Sparks, MD) enabled broth microdilution, processes that required up to 48 h of turnaround. The corresponding refrigerated specimens were tested with DropDx right after selection and required less than 30 min of device operation.

In order to ensure sample freshness, all refrigerated urine specimens were tested in the DropDx platform no more than 1–2 days after collection from the patients. Early in the clinical data validation process, working with urine specimens that contained bacteria‐preserving additives was chosen. Urine specimens at JHH were collected from patients in either a sterile cup or a BD Vacutainer urine collection transport tube (BD Diagnostics, Sparks, MD, #364951). Typically, cup samples do not contain any additives, whereas the much more common Vacutainer tubes include a mixture of boric acid, sodium formate, and sodium borate. Upon confirming that one can observe comparable results from samples without and with the preservatives; additive‐containing specimens were proceeded to be tested predominantly due to their significantly higher abundance and accessibility within the clinical laboratory.

##### Threshold Criteria and Power Analysis for DropDx Clinical Comparison Study

ROC curve and statistical power analyses were implemented to ensure unbiased thresholding and sufficient statistical confidence for data generated. The strategy consisted of using the initial 15 specimens for an AST pilot study, the next 16 specimens concurrently for an ID pilot study and AST validation study, and the final 19 specimens for both ID validation study and AST validation study. Culture‐negative and culture‐positive specimens were differentiated from each other by the total percentage of positive droplets (across FAM and Alexa546 channels) from the no‐antibiotic control (the first DropDx device). EC‐containing specimens were differentiated from non‐EC specimens by the ratio of UNI‐positive droplets to EC‐positive droplets from the first device. Non‐EC EB specimens were differentiated from EB specimens by the percentage of EB‐positive droplets (multiplied by the dilution factor) from the second DropDx device. Importantly, in this case the same normalization (ratio against UNI) used in the no‐antibiotic control was not applied, as it would create inconsistent results for antibiotic‐susceptible and antibiotic‐resistant non‐EB. Finally susceptible specimens were differentiated from resistant specimens by the ratio of UNI‐positive droplets from the second and first DropDx devices. For each category call in the platform (i.e., culture‐negative vs culture‐positive, EC vs non‐EC, EB vs non‐EB, and susceptible vs resistant), ROC curves were plotted using data from each pilot study using GraphPad Prism. Next, an optimal threshold was established by plotting the Youden's Index from each ROC curve, derived by calculating the sensitivity + specificity − 100%, at various ROC thresholds, and then extracting the global maximum value. This threshold was kept constant for all subsequent data collected in the validation studies. Comprehensive raw data from each tested specimen is presented in Table S6 in the Supporting Information.

Power analyses of pilot datasets were conducted by calculating statistical power achieved for varying sample sizes for each category call in MATLAB. The samplesizepwr function was used and the average and standard deviation values from each pilot dataset along with a desired confidence of 95% (*α* = 0.05) were input. Power versus sample size curves were then plotted in MATLAB, followed by a linear interpolation of the number of samples required to reach a power of 90% (1‐*β*).

## Conflict of Interest

The authors declare no conflict of interest.

## Supporting information

Supporting Information

Supplemental Video 1
