# Supplementary material for: Droplet‐Based Single‐Cell Measurements of 16S rRNA Enable Integrated Bacteria Identification and Pheno‐Molecular Antimicrobial Susceptibility Testing from Clinical Samples in 30 min
Source: Adv Sci (Weinh). 2021 Feb 1;8(6):2003419. doi: 10.1002/advs.202003419 (PMC7967084; doi:10.1002/advs.202003419)
Supplement: Supplementary file 1 — Supporting Information [file ADVS-8-2003419-s001.pdf]

WILEY-VCH

© 2020 Wiley-VCH GmbH

ADVANCED SCIENCE
Open Access

Supporting Information

for *Adv. Sci.*, DOI: 10.1002/advs.202003419

Droplet-based single-cell measurements of 16S rRNA enables integrated bacteria identification and pheno-molecular antimicrobial susceptibility testing from clinical samples in 30 min

Aniruddha M. Kaushik, Kuangwen Hsieh,
Kathleen E. Mach, Shawna Lewis, Christopher M. Puleo, Karen C. Carroll,
*Joseph C. Liao, and Tza-Huei Wang**

Supporting Information

Droplet-based single-cell measurements of 16S rRNA enables integrated bacteria identification and pheno-molecular antimicrobial susceptibility testing from clinical samples in 30 min

*Aniruddha M. Kaushik†, Kuangwen Hsieh†, Kathleen E. Mach, Shawna Lewis, Christopher M. Puleo, Karen C. Carroll, Joseph C. Liao, and Tza-Huei Wang**

Summary of supplementary figures and tables:

Figures	Description
Figure S1	**Two-color laser induced fluorescence (LIF) detector and bulk reaction assessment device**
Figure S2	**Bulk pheno-molecular AST of *E. coli* ATCC 25922**
Figure S3	**Modular droplet device for flexible assay characterization**
Figure S4	**One-step sample pretreatment protocol**
Figure S5	**Bulk sensitivity of the PNA probe assay**
Figure S6	**Quantification of antibiotic effect on *E. coli* in droplets**
Figure S7	**Schematic of the entire DropDx platform**
Figure S8	**Thermal platform characterization**
Figure S9	**DropDx device and droplet residence time**
Figure S10	**Validation of pheno-molecular AST in the clinical comparison study**
Figure S11	**Clinical comparison study workflow**
Figure S12	**Pilot studies for power analysis and threshold determination**
**Tables**	**Description**
Table S1	**Summary of designed PNA Probes**
Table S2	**Flow rates for monodisperse droplet generation**
Table S3	**Fluorescence signal of unquenched PNA probes over quenched background**
Table S4	**Summary of urine samples tested and final results in clinical comparison study**
Table S5	**Clinical performance (PPVs and NPVs) of DropDx ID and AST**
Table S6	**Summary of raw droplet data for each sample tested in the clinical comparison study**
**Other**	**Description**
**Video**	**Device video – Picoliter droplet generation and analysis with urine**

Supplementary Figures

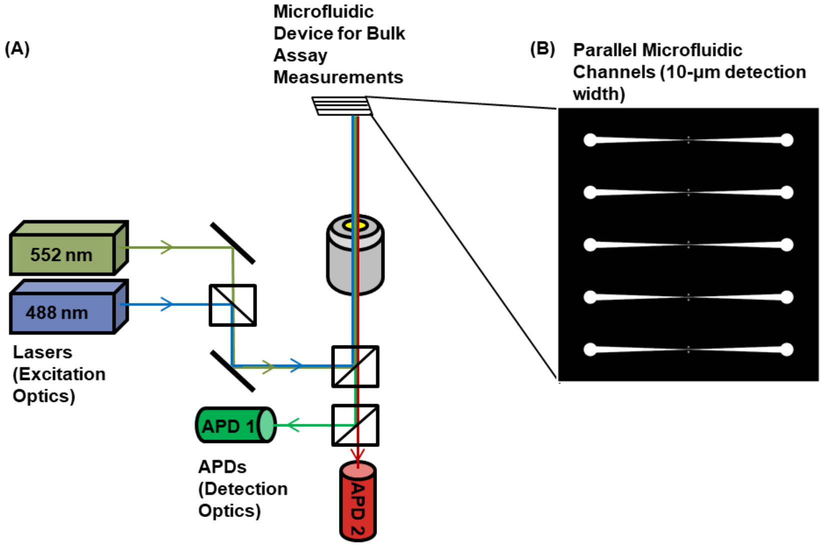

Figure S1. Two-color laser induced fluorescence (LIF) detector and bulk reaction assessment device. (A) Our LIF detector used for both bulk and droplet fluorescence measurements consists of 2 distinct laser illumination sources (488 nm and 552 nm) and 2 avalanche photodiode sensors (APDs 1 & 2). (B) Fluorescence signal for all bulk reactions (used for assay validation and optimization) were measured in custom microfluidic devices that contained parallel channels with 10-μm detection constrictions.

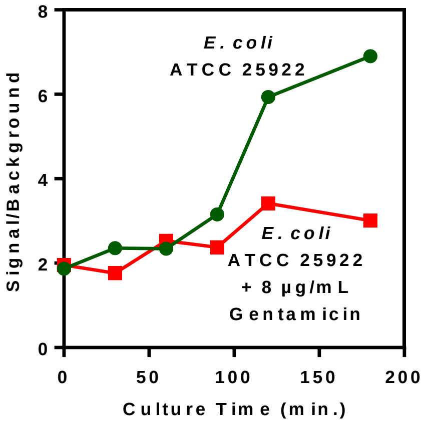

Figure S2. Bulk pheno-molecular AST of *E. coli* 25922. PNA probe signal from *E. coli* cells increases for increasing culture durations, indicative of increased 16S rRNA production as bacteria replicate. In the presence of gentamicin, there is a relatively lower production of 16S rRNA over increasing incubation durations. In bulk, the inhibitory effect of 16S rRNA is noticeable after 90 min of culture.

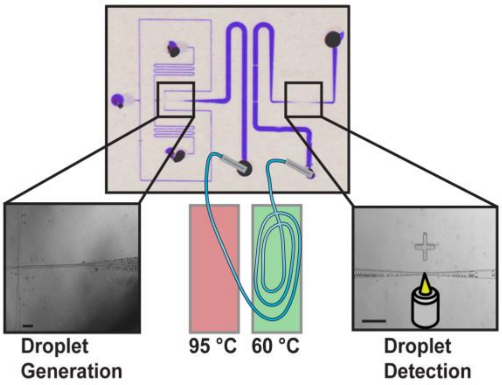

Figure S3. Modular droplet device for flexible assay characterization. A PDMS microfluidic device consisting of a droplet generation region and a normally disconnected droplet detection region served as the platform for assay characterization in droplets. Generated droplets enter into a Tygon tube which traverses over heaters that facilitate bacterial lysis (at 95 °C) and PNA probe hybridization (at 60 °C) before re-entering the device for detection. Hybridization duration of droplets was controlled by varying the length of Tygon tubing that rested on the hybridization heater. Droplet volume was controlled by controlling the height of the channels within in the device. As such, separate devices were used for generation of 1 pL, 4 pL, and 30 pL droplet volumes. Scale bars are ~100 μm.

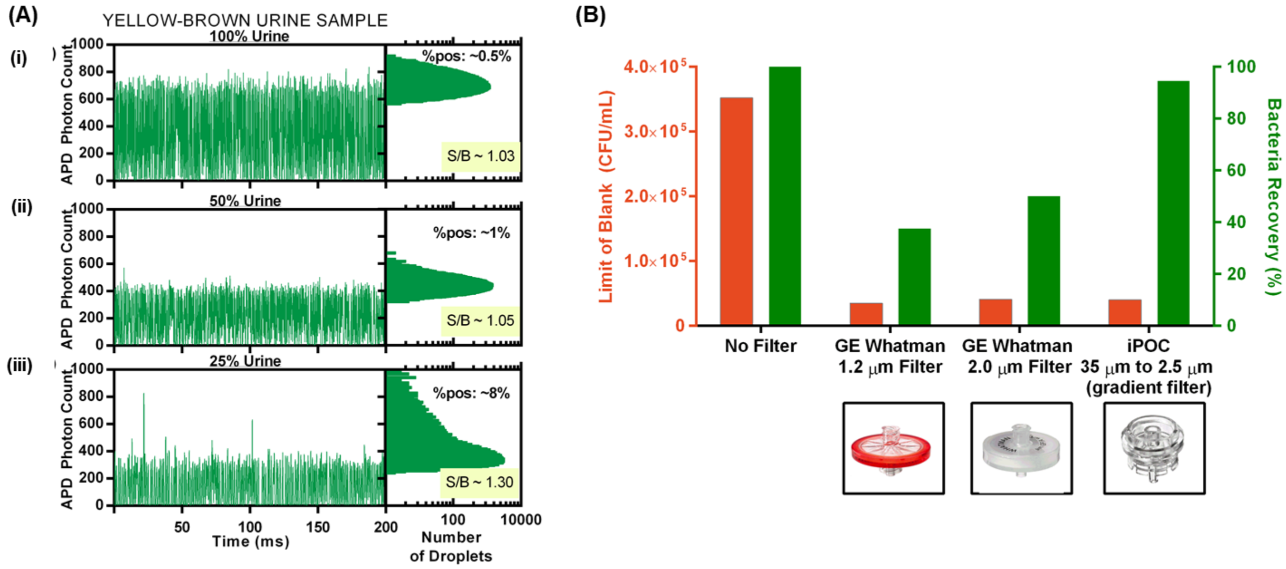

Figure S4. One-step sample pretreatment protocol. (**A**) Our one-step pretreatment protocol includes a single infusion of urine/MH solution through an appropriately sized syringe filter. (**i**) Some urine samples can emit a high auto-fluorescence background. In such cases, (**ii**) 2-fold or (**iii**) or 4-fold dilution of the sample in MH broth is necessary to improve signal to background ratio of positive droplets versus empty droplets, and recover the expected frequency of positive droplets based on the input concentration of bacteria (~10%, here). (**B**) Particulates in urine can impose a high limit of blank (i.e., frequency of empty droplets with high fluorescence intensities from blank/culture-negative urine samples) and hamper bacteria quantification. We tested 3 different syringe filters of varying pore sizes – GE Whatman (pore size 1.2 μm), GE Whatman (pore size: 2.0 μm), and iPOC-Dx Primecare (pore size gradient from 35 μm to 2.5 μm). One-step filtration of the urine/MH mixture through the iPOC-Dx filter reduces limit of blank by more than an order of magnitude, while ensuring up to 94% bacterial recovery for quantification.

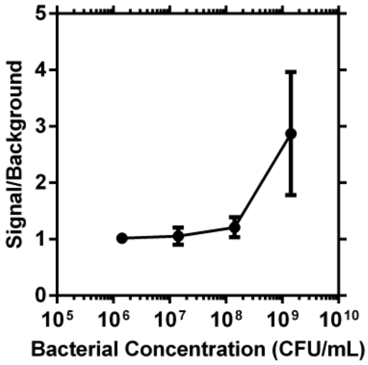

Figure S5. Bulk sensitivity of the PNA probe assay. The signal from bacteria spiked into urine samples were measured and compared to the fluorescence from no-bacteria controls in the same samples. For bulk reactions, at least 1.5 x 10^8 CFU mL^{-1} bacteria must be present in order to effectively measure signal over urine background.

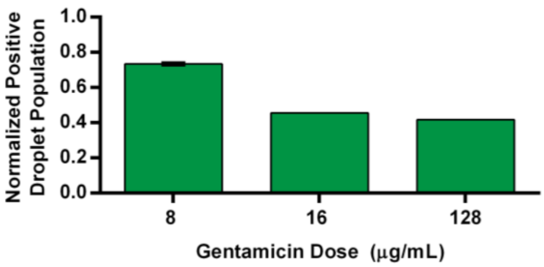

Figure S6. Quantification of antibiotic effect on *E. coli* in droplets. For *E. coli* suspended in MH broth exposed to gentamicin, the normalized positive droplet population decreases as the concentration of gentamicin increases.

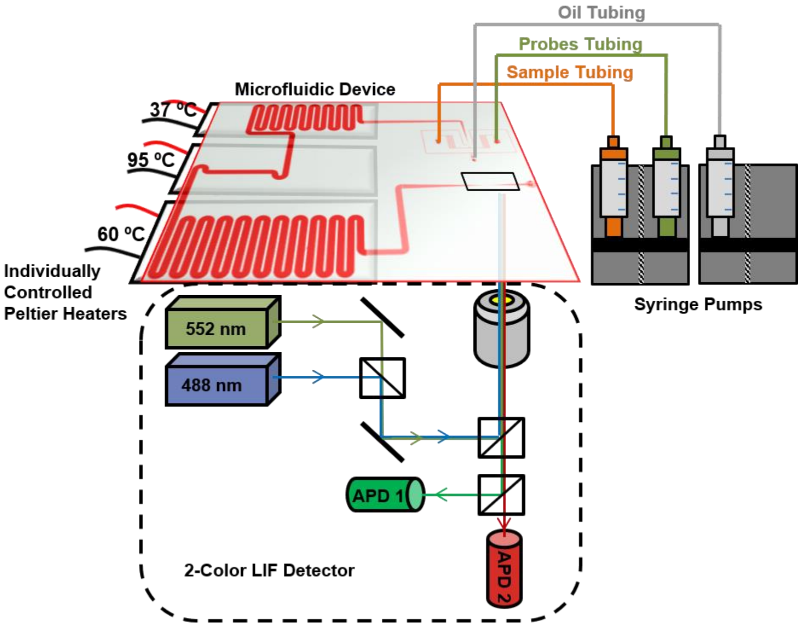

Figure S7. Schematic of the entire DropDx platform (not to scale). The experimental setup consists of a modular (not pictured here) or integrated microfluidic device that rests on individually controlled Peltier heaters. The detection region of the device is aligned to a 2-color LIF detector. Syringe pumps are used to control the flow rates of urine samples, PNA

probes, and droplet generation oil, and are finely tuned to generate stable droplets and propel the droplets through the device for the required incubation durations.

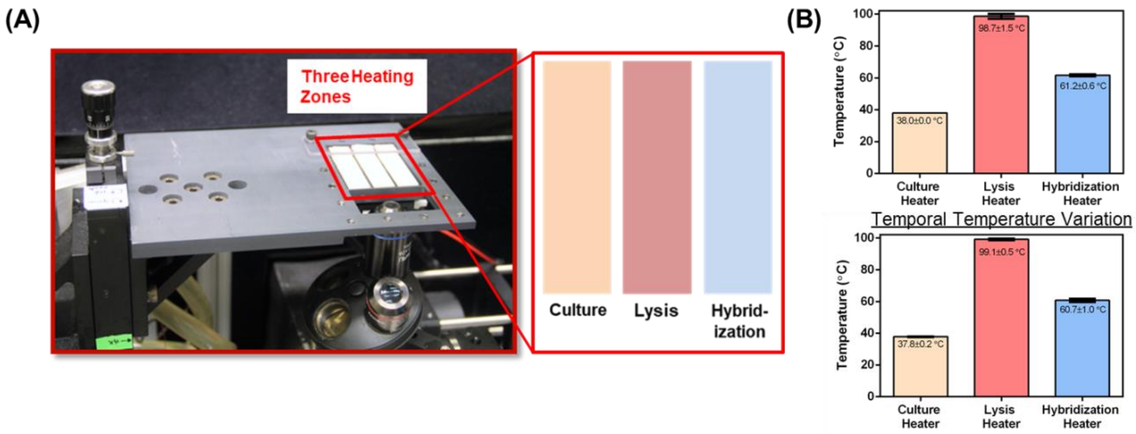

Figure S8. Thermal platform characterization. (**A**) While testing clinical samples, the DropDx device was affixed to a 3-temperature heating rig via thermally conductive paste. (**B**) The thermal rig is able to reliably deliver the correct temperature to the rig with minimal spatial and temporal temperature variation.

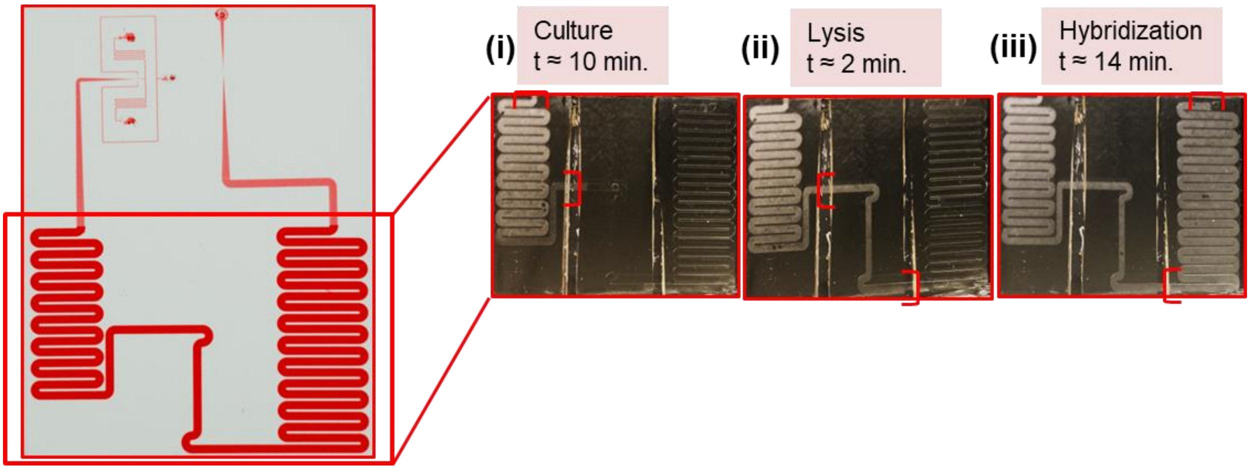

Figure S9. DropDx device and droplet residence time. The residence time of droplets traversing each zone in the droplet device was measured by tracking the front of generated droplets. Droplets spend (**i**) approximately 10 min in the culture/drug exposure zone, (**ii**) approximately 2 min in the lysis zone, and (**iii**) approximately 14 min in the hybridization zone.

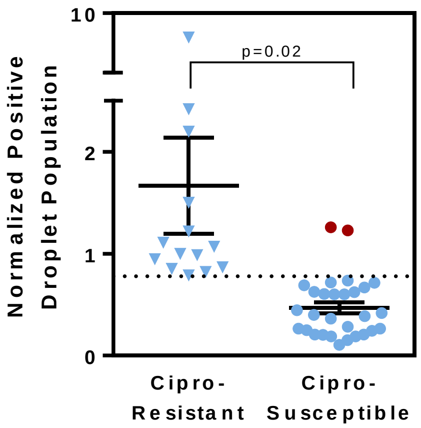

Figure S10. Validation of pheno-molecular AST in the clinical comparison study. Of the 43 culture-positive samples tested, DropDx can accurately differentiate ciprofloxacin-resistant from ciprofloxacin-susceptible samples (p = 0.02), resulting in a 95.3% categorical agreement with the clinical standard methodology.

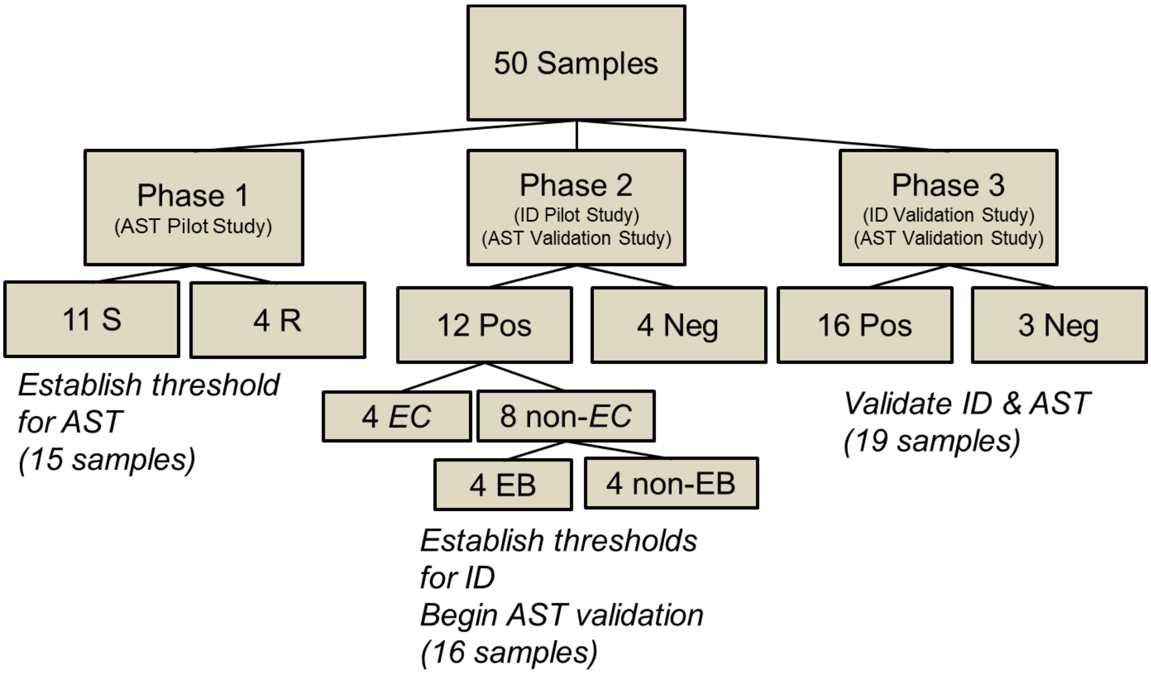

Figure S11. Clinical comparison study workflow. Data collection was divided into 3 phases. Phase 1, "AST Pilot Study" included the first 15 urine samples tested. Pilot studies were used to establish a data-agnostic threshold for susceptibility/resistance calls and determine the minimum number of samples required for adequate statistical power. Phase 2, "ID Validation Study" included the next 16 samples tested and was used to set up thresholds for the ID classification categories used in this workflow. Phase 2 was also used to validate our measurements for susceptibility/resistance. The final set of samples was used to validate both ID classification categories as well as susceptibility/resistance.

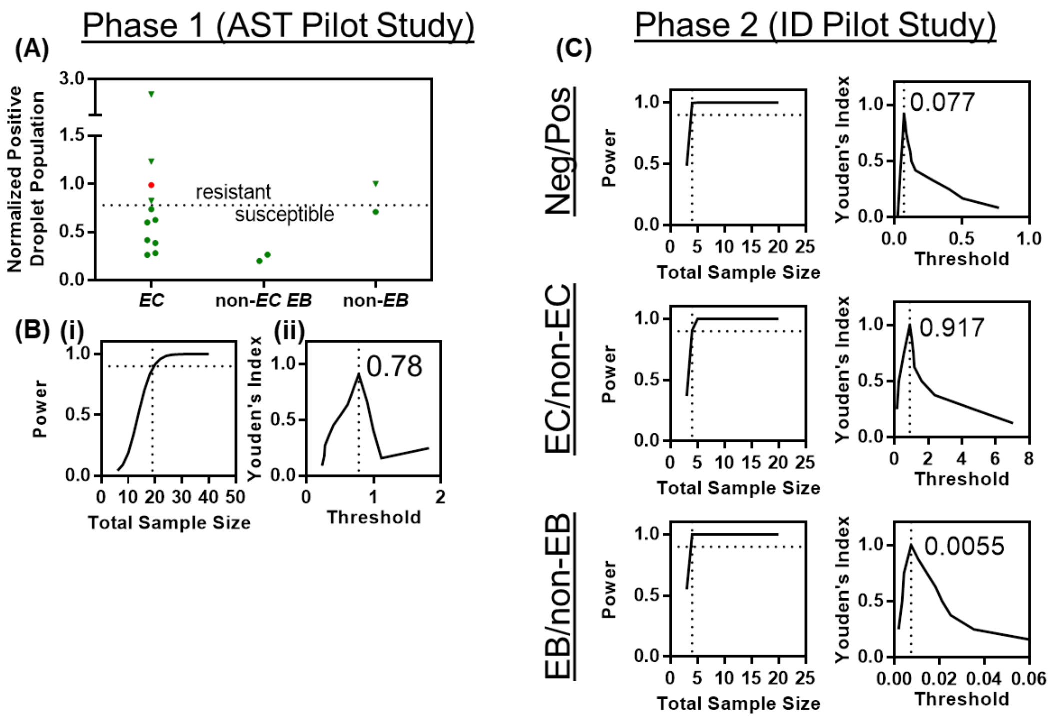

Figure S12. Pilot studies for power analysis and threshold determination. (A) Data collected in Phase 1, the AST Pilot Study, was plotted for each category of sample interrogated. Ciprofloxacin-resistant samples (represented by inverted triangles) are well separated from ciprofloxacin-susceptible samples (represented by circles), with 1 false-resistant call. (**B**) (**i**) Power analysis was conducted using the pilot study data to determine that at least 20 samples must be interrogated in the validation phase to ensure a statistical power (1-β) of 90% and a confidence (α) of 95%. (**ii**) ROC analysis of the pilot study data was used to determine a data-agnostic threshold that maximizes the Youden's Index of the pilot dataset. This threshold was kept constant for all subsequent data analyzed. (**C**) Power analyses and ROC-based threshold determination was repeated in Phase 2, the ID Pilot Study, for each classification criteria utilized in DropDx.

Supplementary Tables

Table S1. Summary of designed PNA Probes. We designed and tested 4 unique PNA probes, specific to the species *E. coli* and *P. mirabilis*, the *Enterobacterales* order, and the bacterial kingdom (eubacteria). Custom DNA quenchers were designed tagged with an Iowa-Black quencher that spanned 10-11 complimentary bases to the PNA probes.

Probe	Target	Sequence	T_m (°C)
EC	*E. coli*	FAM-O-TCAATGAGCAAAGGT-KK<br>IABk-AGTTACTCGT	76.3
PM	*P. mirabilis*	FAM-O-TCCTCTATCTCTAAAGG<br>IAbk-AGGAGATAGAG	67.2
**EB**	Enterobacterales	FAM-O-TATGAGGTCCGCTTG<br>IABk-ATACTCCAGG	73.4
**UNI**	Eubacteria	GCTGCCTCCCGTAGGA-K-Alexa546<br>GGAGGGCATCCT-IABk	80.5

Table S2: Flow rates for monodisperse droplet generation. The flow rate of the continuous phase oil was fixed at 60 μL h^{-1}. The effect of the flow rates of pretreated urine and hybridization buffer on droplet diameter and polydispersity was measured. Droplet diameter was acquired from micrographs of generated droplets using ImageJ. The polydipsersity index is the ratio of the standard deviation to the average of measured droplet diameters. The selected flow rate for all experiments in this work is highlighted in green.

Oil Flow Rate (μL h^{-1})	Urine & Hybridization Buffer Flow Rate (μL h^{-1})	Average Droplet Diameter (μm)	Polydispersity Index (CV of Droplet Diameter)
60	> 25	NA	NA
60	25	22	8.6%
60	20	21	7.5%
60	15	20	3.2%
60	10	21	4.9%
60	5	22	10.1%
60	2	23	5.1%
60	< 2	NA	NA

Table S3: Fluorescence signal of unquenched PNA probes over quenched background. The selected laser power for all experiments in this work is highlighted in green. Bulk-based measurements acquired using LIF detector.

488 nM laser line for detection of FAM-labeled PNA probes			
Power (mW)	Quenched probe fluorescence intensity (APD Photon Count)	Unquenched probe fluorescence intensity (APD Photon Count)	Signal/Background
2	63.4	81.1	1.279179811
4	106	144	1.358490566
6	150.2	205	1.364846871
8	193.7	266	1.373257615
10	212.3	322	1.51672162
552 nM laser line for detection of Alexa546-labeled PNA probes			
Power (mW)	Quenched probe fluorescence intensity (APD Photon Count)	Unquenched probe fluorescence intensity (APD Photon Count)	Signal/Background
2	213	510	2.394366197
4	362	925	2.555248619
6	514	1340	2.607003891
8	636	1750	2.751572327
10	778	2130	2.737789203

Table. S4. Summary of urine samples tested and final results in clinical comparison study. For each sample tested, a summary of the ID and AST readouts from clinical standard workflows (MALDI/TOF and BD Phoenix) is listed against the DropDx classification and resistance call. Disagreements in data are highlighted in red.

		Clinical Readout		DropDx Readout		
**Internal Designation**	**Sample Number**	**Pathogen ID**	**Ciprofloxacin Resistance**	**Pathogen ID**	**Ciprofloxacin Resistance**	**Notes**
BA2-10182018	1	*E. coli*	S	NA	S	AST Pilot
BA5-10232018	2	*K. pneumoniae*	S	NA	S	AST Pilot
BA6-10232018	3	*E. coli*	S	NA	S	AST Pilot
BA7-10232018	4	*E. coli*	S	NA	R	AST Pilot
BA8-10232018	5	*P. vulgaris*	S	NA	S	AST Pilot
BA9-10302018	6	*E. coli*	S	NA	S	AST Pilot
BA14-11012018	7	*E. coli*	S	NA	S	AST Pilot
BA15-11012018	8	*E. coli*	S	NA	S	AST Pilot
BA16-11012018	9	*E. coli*	R	NA	R	AST Pilot
BA17-11012018	10	*E. coli*	R	NA	R	AST Pilot
BA18-11072018	11	*E. coli*	S	NA	S	AST Pilot
BA19-11072018	12	*E. coli*	R	NA	R	AST Pilot
BA21-11072018	13	*P. aeruginosa*	S	NA	S	AST Pilot
BA22-11272018	14	*P. aeruginosa*	R	NA	R	AST Pilot
BA23-11272018	15	*E. coli*	S	NA	S	AST Pilot
BA26-12042018	16	*K. pneumoniae*	S	non-*EC EB*	S	ID Pilot/ AST Validation
BA29-12042018	17	*E. coli*	S	*EC*	S	ID Pilot/ AST Validation
CC30-12122018	18	*S. marsescens*	S	non-*EC EB*	S	ID Pilot/ AST Validation
BA31-12112018	19	*E. coli*	S	*EC*	S	ID Pilot/ AST Validation

BA34-12112018	20	Negative	NA	Negative	NA	ID Pilot/ AST Validation
BA38-12132018	21	*E. cloacae*	S	non-*EC EB*	S	ID Pilot/ AST Validation
BA41-12132018	22	Negative	NA	Negative	NA	ID Pilot/ AST Validation
BA42-12182018	23	*E. coli*	R	*EC*	R	ID Pilot/ AST Validation
BA48-12202018	24	*K. oxytoca/ R. orinthinolytica*	S	non-*EC EB*	S	ID Pilot/ AST Validation
BA49-12202018	25	*E. coli*	S	*EC*	S	ID Pilot/ AST Validation
BA58-01032019	26	Negative	NA	Negative	NA	ID Pilot/ AST Validation
BA63-01102019	27	Negative	NA	Negative	NA	ID Pilot/ AST Validation
BA85-02192019	28	*P. aeruginosa*	S	non-*EB*	S	ID Pilot/ AST Validation
BA86-02192019	29	*P. aeruginosa*	S	Negative	R	ID Pilot/ AST Validation
BA90-02142019	30	*P. aeruginosa*	S	non-*EB*	S	ID Pilot/ AST Validation
BA54-01032019	31	*P. aeruginosa*	S	non-*EB*	S	ID Pilot/ AST Validation
BA57-01032019	32	*K. pneumoniae*	S	non-*EC EB*	S	Validation
BA60-01102019	33	*E. coli*	S	*EC*	S	Validation
BA61-01102019	34	*E. coli*	S	*EC*	S	Validation
BA64-01102019	35	Negative	NA	Negative	NA	Validation
BA68-01172019	36	Negative	NA	Negative	NA	Validation
BA76-01242019	37	Negative	NA	Negative	NA	Validation
BA91-02142019	38	*P. aeruginosa*	S	non-*EB*	S	Validation
BA92-02212019	39	*P. aeruginosa*	S	non-*EB*	S	Validation
BA93-02212019	40	*P. aeruginosa*	S	non-*EB*	S	Validation
BA97-03142019	41	*P. mirabilis*	R	non-*EC EB*	R	Validation
BA98-03282019	42	*P. aeruginosa*	R	non-*EC EB*	R	Validation
BA100-03282019	43	*E. coli*	R	*EC*	R	Validation
BA101-04042019	44	*E. coli*	R	*EC*	R	Validation
BA104-04112019	45	*E. coli*	R	*EC*	R	Validation
BA107-05072019	46	*E. coli*	R	*EC*	R	Validation
BA108-05072019	47	*E. coli*	R	*EC*	R	Validation
BA111-05282019	48	*E. coli*	R	*EC*	R	Validation
BA112-05312019	49	*C. freundii*	S	non-*EC EB*	S	Validation
BA113-05312019	50	*E. coli*	R	*EC*	R	Validation

Table. S5. Clinical performance (PPVs and NPVs) of DropDx ID and AST. The true positive, false positive, true negative, fasle negative, PPVs, and NPVs are tabulated for DropDx readouts for (A) culture negative/positive, (B) *EC* negative/positive, (C) *EB* negative/positive, and (D) ciprofloxacin resistant/susceptible metrics.

(A) **Standard Clinical Readout**

(B) **Standard Clinical Readout**

	Culture Positive	Culture Negative
**DropDx Culture Positive**	27	0
**DropDx Culture Negative**	1	7
	**PPV: 27/28 = 96.4%**	**NPV: 7/7 = 100%**

	EC Positive	*EC* Negative
**DropDx *EC* Positive**	13	0
**DropDx *EC* Negative**	0	15
	**PPV: 13/13 = 100%**	**NPV: 15/15 = 100%**

(C)	Standard Clinical Readout	
	***EB* Positive**	***EB* Negative**
**DropDx *EB* Positive**	20	1
**DropDx *EB* Negative**	0	7
	**PPV: 20/20 = 100%**	**NPV: 7/8 = 87.5%**

(D)	Standard Clinical Readout	
	**Cipro Resistant**	**Cipro Susceptible**
**DropDx Cipro Resistant**	14	2
**DropDx Cipro Susceptible**	0	27
	**PPV: 14/14 = 100%**	**NPV: 27/29 = 93.1%**

Table. S6. Summary of raw droplet data for each sample tested in the clinical comparison study.

Internal Designation	Appearance	Fold Dilution	Total Droplets Interrogated	UNI % Positive	EC % Positive	EB % Positive	UNI % Positive (Cipro)	UNI/EC	Normalized Positive Droplet Population (Resistance)
BA2-10182018	Light orange to clear	4	729169	0.0988	NA	NA	0.0728	NA	0.7368
BA5-10232018	Light yellow-brown	10	1564831	0.2300	NA	NA	0.0464	NA	0.2017
BA6-10232018	Light yellow-brown to clear	4	1230390	0.0539	NA	NA	0.0324	NA	0.6011
BA7-10232018	Light yellow-brown	10	474937	0.1300	NA	NA	0.1600	NA	1.2308
BA8-10232018	Dark yellow-brown	10	683206	0.1200	NA	NA	0.0319	NA	0.2658
BA9-10302018	Light yellow-brown	10	935450	0.4800	NA	NA	0.2000	NA	0.4167
BA14-11012018	Light yellow-brown	4	1251537	0.1700	NA	NA	0.0480	NA	0.2824
BA15-11012018	Light brown to clear	4	1496388	0.1500	NA	NA	0.0396	NA	0.2640
BA16-11012018	Light brown to clear	4	714160	0.0779	NA	NA	0.0641	NA	0.8228
BA17-11012018	Yellow, slightly turbid	10	903881	0.0826	NA	NA	0.2000	NA	2.4213
BA18-11072018	Light brown, turbid	10	931415	0.1600	NA	NA	0.1000	NA	0.6250
BA19-11072018	Brown	10	1690939	0.0855	NA	NA	0.0845	NA	0.9883
BA21-11072018	Light yellow to clear	4	763975	0.0283	NA	NA	0.0195	NA	0.6890
BA22-11272018	Yellow, slightly turbid	4	1232669	0.0399	NA	NA	0.0400	NA	1.0025

BA23-11272018	Bright yellow	10	602634	0.0956	NA	NA	0.0370	NA	0.3870
BA26-12042018	Light yellow	4	1829478	0.1100	0.0149	0.0047	0.0439	7.3826	0.3991
BA29-12042018	Light yellow	4	1705296	0.1000	0.3400	0.0060	0.0605	0.2941	0.6050
CC30-12122018	Light to clear	4	1203970	0.1100	0.0164	0.0027	0.0165	6.7073	0.1500
BA31-12112018	Yellow	10	1727534	0.0338	0.1500	0.0095	0.0243	0.2253	0.7189
BA34-12112018	Yellow	10	887857	0.0103	0.0425	NA	NA	NA	NA
BA38-12132018	Light to clear	4	1331620	0.6600	0.3200	0.0026	0.0704	2.0625	0.1067
BA41-12132018	Light yellow	4	503231	0.0221	0.0072	NA	NA	NA	NA
BA42-12182018	Light yellow-brown	4	513048	0.0408	0.5300	0.0045	0.0898	0.0770	2.2010
BA48-12202018	Light yellow	4	976234	0.2000	0.1700	0.0065	0.1200	1.1765	0.6000
BA49-12202018	Light pink	7	1272413	0.0503	0.0595	0.0064	0.0337	0.8454	0.6700
BA58-01032019	Light white to clear	4	971632	0.0303	0.0044	NA	NA	NA	NA
BA63-01102019	Yellow	4	310989	0.0460	0.0193	NA	NA	NA	NA
BA85-02192019	Light to clear	4	1173232	0.0418	0.0357	0.0007	0.0079	1.1709	0.1895
BA86-02192019	Light yellow to clear	4	729830	0.0073	0.0068	0.0011	0.0092	1.0800	1.2606
BA90-02142019	Yellow	4	1050350	0.0425	0.0430	0.0002	0.0089	0.9884	0.2085
BA54-01032019	Light yellow	4	843963	0.0659	0.0241	0.0010	0.0164	2.7344	0.2489
BA57-01032019	Light to clear	4	728876	0.2100	0.0994	0.0045	0.1500	2.1127	0.7143
BA60-01102019	Yellow	10	1015568	0.0795	0.0903	0.0020	0.0194	0.8804	0.2440
BA61-01102019	Yellow	10	1636813	0.0173	0.6000	0.0014	0.0108	0.0288	0.6243
BA64-01102019	Yellow-brown	10	600147	0.0707	0.0058	NA	NA	NA	NA
BA68-01172019	Light yellow	4	627665	0.0300	0.0061	NA	NA	NA	NA
BA76-01242019	Yellow	4	841261	0.0263	0.0109	NA	NA	NA	NA
BA91-02142019	Yellow	4	602600	0.0749	0.0266	0.0008	0.0154	2.8158	0.2056
BA92-02212019	Yellow-brown	4	992221	0.0909	0.0285	0.0012	0.0172	3.1895	0.1892
BA93-02212019	Yellow-brown	4	1604698	0.1800	0.0820	0.0012	0.0799	2.1951	0.4439
BA97-03142019	Yellow to light brown	4	789165	0.0849	0.0084	0.0077	0.0940	10.0951	1.1072
BA98-03282019	Yellow	4	404895	0.6100	0.0474	0.0045	0.5300	12.8692	0.8689
BA100-03282019	Yellow to clear	4	468131	0.0319	0.0562	0.0014	0.0478	0.5676	1.4984
BA101-04042019	Yellow	4	556509	0.3400	0.3900	0.0934	0.2900	0.8718	0.8529
BA104-04112019	Light yellow	4	635381	0.5200	4.6300	0.0085	0.4100	0.1123	0.7885
BA107-05072019	Brown	4	826703	0.0185	0.0789	0.0025	0.1400	0.2345	7.5676
BA108-05072019	Light yellow-brown	4	1119261	0.0051	3.5100	0.0021	0.0063	0.0015	1.2203
BA111-05282019	Light brown to clear	4	385768	0.1500	0.6500	0.1200	0.1600	0.2308	1.0667
BA112-05312019	Light yellow to turbid	4	468032	0.1200	0.0233	0.0019	0.0436	5.1502	0.3633
BA113-05312019	Light yellow-brown	4	503236	0.0441	9.7300	0.0052	0.0417	0.0045	0.9456
